# Single-cell transcriptomics reveals reversible microglia/macrophage activation after human retinal neuron transplantation into mouse retina

**DOI:** 10.1016/j.isci.2026.117232

**Published:** 2026-10-21

**Authors:** Emil Kriukov, Anthony Mukwaya, Paul Francis Cullen, Hio Tong Kam, George Baldwin, Volha V. Malechka, Nasrin Refaian, Nikita Bagaev, Everett Labrecque, Sthavir Vinjamuri, Dong Feng Chen, Milica A. Margeta, Petr Baranov

**Affiliations:** 1 Schepens Eye Research Institute of Massachusetts Eye and Ear, Boston, MA, USA; 2 Department of Ophthalmology, Harvard Medical School, Boston, MA, USA; 3 Department of Ophthalmology, University of Pittsburgh, Pittsburgh, PA, USA

**Keywords:** retina, microglia, single-cell RNA sequencing, transplantation, cell fate, activation, inflammation

## Abstract

Host retinal microglia and macrophages can limit donor neuron survival and integration after transplantation, but the cellular mechanisms underlying this response remain unclear. In this study, we used human embryonic stem cell-derived retinal ganglion cells, generated from Brn3b-tdTomato-Thy1.2-derived retinal organoids, and transplanted them subretinally into healthy adult CX3CR1^GFP^ mice. Three days later, GFP-positive host myeloid cells were isolated by fluorescent activated cell sorting and profiled using 10× single-cell RNA sequencing, with immunohistochemistry validating spatial and molecular changes. Transcriptomic and RNA velocity analyses revealed a microglia/macrophage activation continuum, including homeostatic, transitory, proliferative, and activated states. Transplantation induced a disease-associated microglia-like program, marked by reduced Tmem119 and increased Apoe, Lgals3, Spp1, Cybb, and related activation genes. Integrated atlas comparisons linked this response to retinal and neurodegenerative disease-associated myeloid states. Importantly, separate homeostatic-to-activated and activated-to-homeostatic trajectories suggest that transplantation-induced host myeloid activation is dynamic and partially reversible, with implications for improving future retinal cell therapies.

## Highlights

• Host myeloid cells form dynamic states after human RGC transplantation
• Transplantation induces a DAM-like Apoe/Spp1/Lgals3 activation program
• RNA velocity reveals bidirectional microglia activation and recovery trajectories
• Cell commitment can be studied using RNA velocity output

## Graphical abstract

**Figure:**
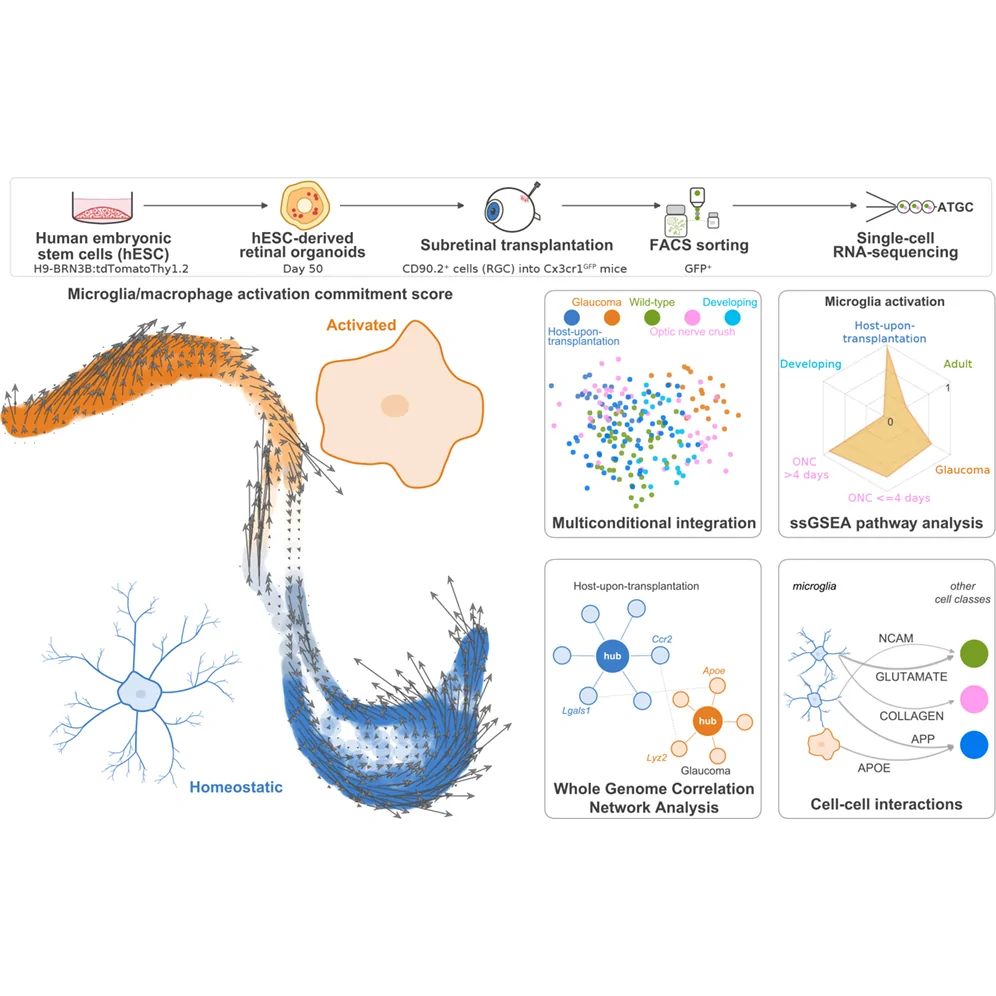

## Teaser

Neuroscience; Cellular neuroscience; Transcriptomics

## Introduction

Retinal ganglion cells (RGC) play a critical role in the transmission of visual signals from the retina to the brain. The optic neuropathies, including glaucoma, non-arteritic anterior ischemic optic neuropathy, Leber Hereditary Optic Neuropathy, traumatic optic neuropathy, and optic pathway glioma result in damage to RGC, ultimately leading to their death. RGC loss is permanent given that these cells cannot regenerate in mammals.^1^ Neuroregenerative strategies for targeting vision loss in optic neuropathies have been mainly focused on restoring the lost RGC cell population in a glaucomatous retina. This approach not only aims to halt disease progression, but also to restore the lost vision by replacing the damaged/lost RGC through RGC transplantation.^2^^,^^3^^,^^4^ To achieve functional regeneration, the transplanted RGC must survive, integrate, and extend their axons and establish connections within the retina and the brain. Of the many factors influencing initial donor RGC survival,^2^^,^^4^^,^^5^^,^^6^^,^^7^^,^^8^^,^^9^ the innate immunity^10^ and primarily microglia reactivity^11^^,^^12^ are of particular interest. In animal models of glaucoma and other optic neuropathies, microglia have been heavily implicated in driving disease progression, promoting a pro-inflammatory environment.^13^^,^^14^ In some instances, activated microglia have been directly implicated in RGC loss by mediating phagocytosis of the damaged or injured RGC.^15^ In line with this, our recent work shows that activated host microglia are detrimental to donor RGC survival, presumably via phagocytosis of the donor RGC. We observed that pre-treatment of donor RGC with sFasL and Annexin V to block the exposed phosphatidylserine residues of the stressed donor RGC prior to transplantation reduced activation of host microglia and resulted in better RGC survival rates post transplantation.^16^ Studies like these and others underscore the importance of modulating host microglia reactivity for better transplantation outcomes.

To further improve donor RGC survival after transplantation, a better understanding of the host myeloid cell reactivity following donor RGC transplantation is paramount. In the current study, we profiled the transcriptome of the host myeloid (*CX3CR1*^*GFP*^) cell population using a single-cell RNA sequencing approach, to delineate the myeloid cell responses on a single-cell level. A focused analysis of the microglial cell population was performed, along with the comparison to microglia from healthy adult mouse retina cell atlas we have assembled in this study. We demonstrate that microglia following RGC transplantation upregulate disease associated microglia (DAM) genes, including *Apoe*, *Spp1*, and *Lgals3*, which are classically seen in neurodegenerative diseases of the brain and the retina.^17^^,^^18^^,^^19^ In addition, we identified several other genes associated with microglia activation, including *Cybb*, *Atf3*, *Aurka*, and *Kif22*. Given the known function of these genes in other disease contexts, we believe that these genes are important for modulating donor RGC survival post transplantation and thus, warrant further investigation in this context. Taken together, we present a comprehensive analysis of the host myeloid cell population following transplantation at a single-cell resolution level, specifically focusing on microglia reactivity, and provide insights into the regulatory mechanisms that may be important for donor RGC survival and integration into injured retina.

## Results

### Healthy adult mouse retina atlas allows for reference-based studies of a defined class of retinal cells in young and aged mice

We sourced, analyzed, and integrated multiple publicly available single-cell RNA sequencing datasets from healthy adult mouse retina, establishing a “healthy adult mouse retina atlas” which is now available in the web-browser format (https://cellxgene.cziscience.com/e/3375d9f3-e17b-46da-b40e-ec6e1779ad88.cxg/). The atlas contains a total of 373,651 cells, from postnatal week 4 to 52-week-old mice (Figures 1A and S2). Using canonical cell markers, major retinal cell classes were identified in the atlas. These include RGC, amacrine cells (AC), horizontal cells (HC), bipolar, rod and cone photoreceptor, Müller glia, immune/myeloid/lymphoid cells, retinal pigment epithelial cells (RPE), and vascular endothelial cells based on the expression of their respective known cell class-specific genes such as Tfap2a, Tfap2b, Onecut2, Grik1, Vsx1, Vsx2, Pde6h, Cx3cr1, Tmem119, Rlbp1, Gap43, Rbpms, Pou4f2, Isl1, Nrl, Nr2e3, Pde6b, Rcvrn, Rpe65, Emcn, and Pdgfrb, respectively (Figures 1A–1C).

**Figure 1:**
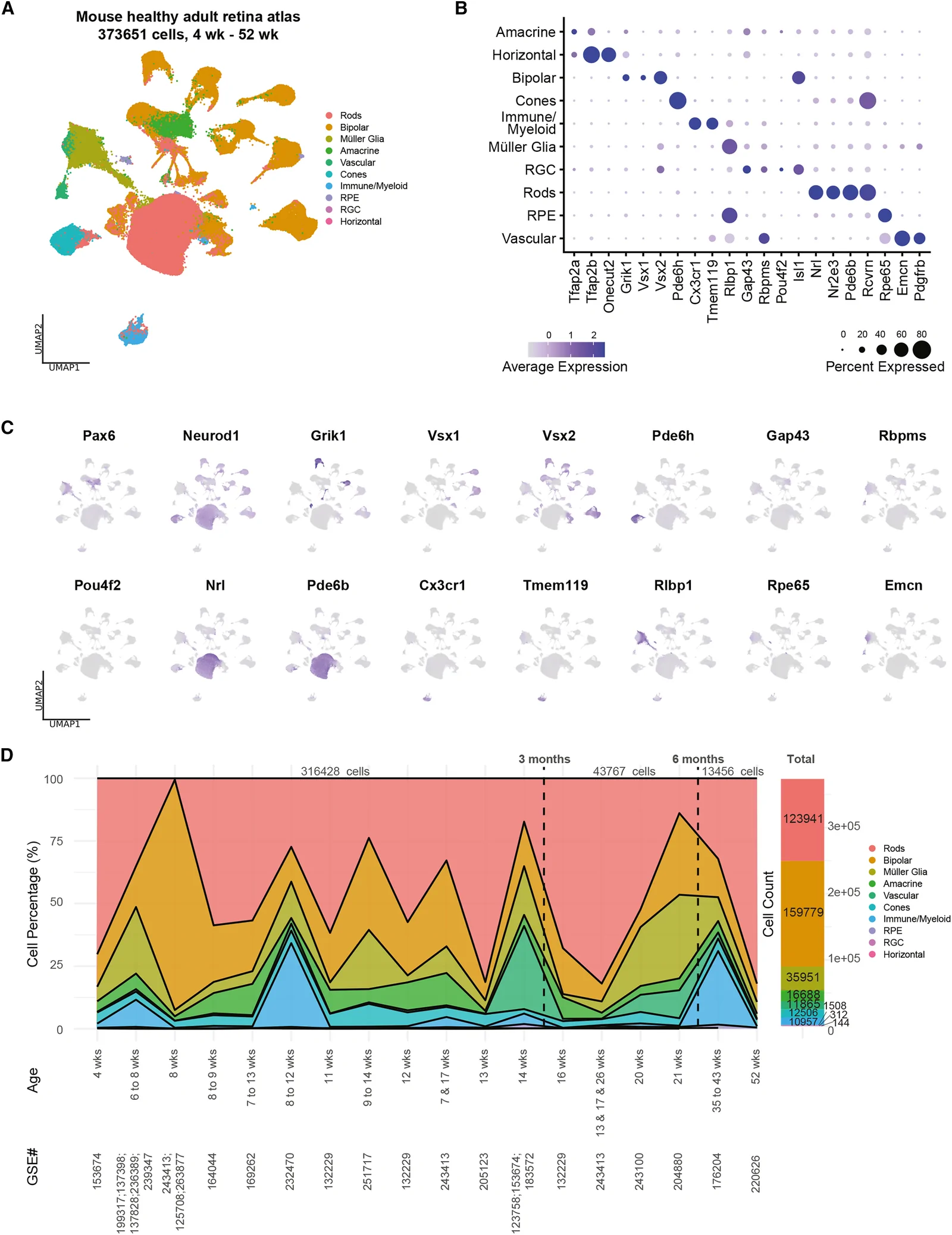
Mouse healthy adult retina atlas demonstrates high resolution of retinal cell classes over a broad timeline (A) UMAP embedding of mouse healthy retina atlas (373,651 cells) highlighting the major retinal cell classes identified. (B) Dot plot presenting the panel of canonical markers for the cell classes identified in the atlas. (C) Feature plots highlighting the cells expressing the class-specific markers. (D) Cumulative chart showing the atlas composition for the relative and absolute number of cells, time points distribution, and the relevant public dataset identifiers.

When the integrated data were stratified by biological timepoints (Figure 1D), the technical composition of different cell classes varied at the different time points. Rods for instance constituted the largest percentage of the retinal neurons throughout the timeline. For a more streamlined downstream analysis, the data in the atlas was reclassified generating three major time frames (Figure S2).

• Young adult (≤14 weeks old).
• Mature adult (15 weeks–21 weeks old).
• Aged adult (>35 to 43 weeks and 52 weeks old).

This reclassification maintained a sufficient resolution to define different cell classes, and time point for in-depth subset population-oriented studies, and for cell-cell interaction analysis (Figure S3). The major cell classes were maintained even after the reclassification. The immune/myeloid/lymphoid cell populations contained 10,957 cells across the timeline: 9,979, 205, and 773 in young adult, mature adult, and aged adult, respectively. The 10,957 of the immune/myeloid/lymphoid cells represent 46 separate batches (Figure 2A). To organize the immune/myeloid/lymphoid compartment within the integrated adult retina atlas and to identify transcriptionally distinct subpopulations and their marker programs, we perform cell fate trajectory reconstruction (Figure 2B). The resulting trajectory indicates two potential cell fate bifurcations, which in turn resulted in three final cell subpopulations including dendritic cells, T cells, and microglia/macrophages (Figure 2C). The first bifurcation, defined by Cxcl10+ Ccl2+ Tnf+, separates immune cells and *microglia/macrophages defined by Il1b+ Cybb+ Lyz2*. The second bifurcation, present within the immune cell population, further subdivides into dendritic cells (Ccr7+) and T cells (Cd3e+ Cd3g+ Ccl5+ Il1b+) (Figure 2D). This allowed us to define the populations and their exclusive markers within the immune/myeloid/lymphoid population on the healthy adult mouse retina atlas for further reference mapping of the host-upon-transplantation microglia dataset.

**Figure 2:**
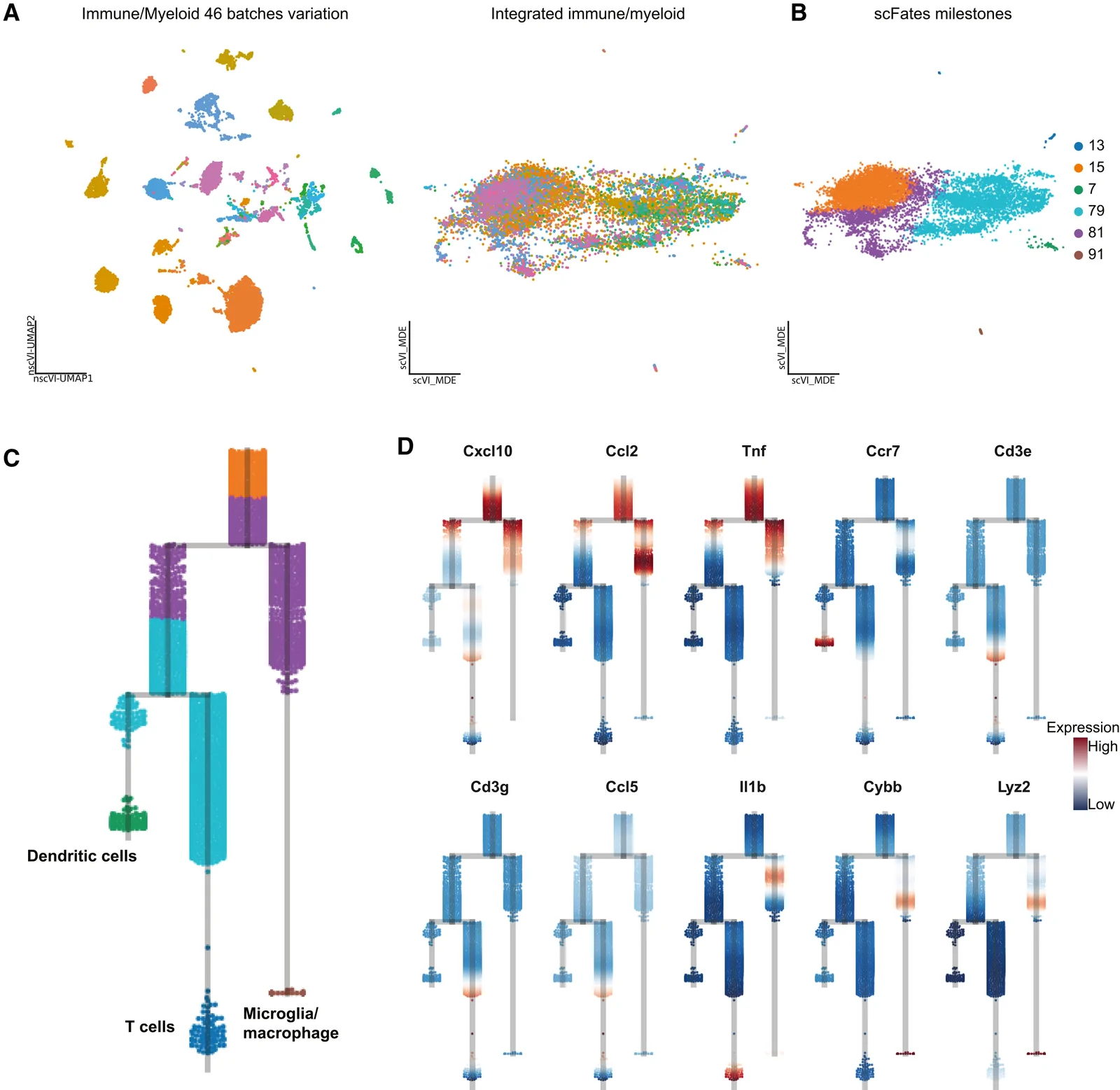
Healthy adult retina myeloid subset cell fate trajectory inference analysis decomposes the population into the major cell types (A) Healthy adult retina myeloid subset (46 batches, where each batch is treated as an individual SRR) upon scVI reembedding. The left embedding demonstrates significant batch variation that was adjusted on the right plot of integrated myeloid. (B) Major milestones, or clusters, present in the myeloid subset upon scFates analysis. (C) Dendrogram highlighting the trajectory of milestones. Milestone 15 gives rise to two branches, with the left split into dendritic cells and T cells, while the right is true microglia/macrophage. (D) Dendrograms demonstrating the expression of branch-related genes, classically known for the dendritic, T, and microglia/macrophage cells.

### Host microglia respond to RGC transplantation with a continuous and reversible cell activation state

Following RGC transplantation, the host retina myeloid cell population was isolated and used for single-cell RNA sequencing (Figure S1). A total of 2,814 cells were recovered after filtering and quality control. To define microglia/macrophage cells in the host myeloid dataset, automated cell annotation was performed using the previously established adult mouse retina atlas (available here: https://cellxgene.cziscience.com/e/3375d9f3-e17b-46da-b40e-ec6e1779ad88.cxg/) as the reference atlas. Upon subsetting the microglia/macrophage cells, trajectory reconstruction of the annotated cells using RNA velocity indicated a continuum of cell state upon embedding, as demonstrated by the velocity pseudotime (Figure 3A). In total three myeloid cell states (homeostatic, proliferating, and activated, based on their expression profile) were identified i.e., the two ends referred to as the “*tip*” states (top left and bottom right ends of the trajectory) and the “*transitory*” state (the in-between the two tips), highlighted with Mellon density plot (Figure 3A). The trajectory, built in an unsupervised manner, demonstrates a continuous connection between the two distinct cell states of microglia/macrophage cells i.e., homeostatic and activated. We observed a bidirectional commitment of cells. The cells in the homeostatic state could transition into an activated state, while cells in the activated state could transition back to the homeostatic state. Importantly, these trajectories are determined by different transcriptional programs, with no major overlap on the embedding. The homeostatic and activated cell states representing the top and bottom ends of the pseudotime, respectively were defined using the homeostatic and activated scores based on known genes associated with microglial^20^ states (Figure 3B).

**Figure 3:**
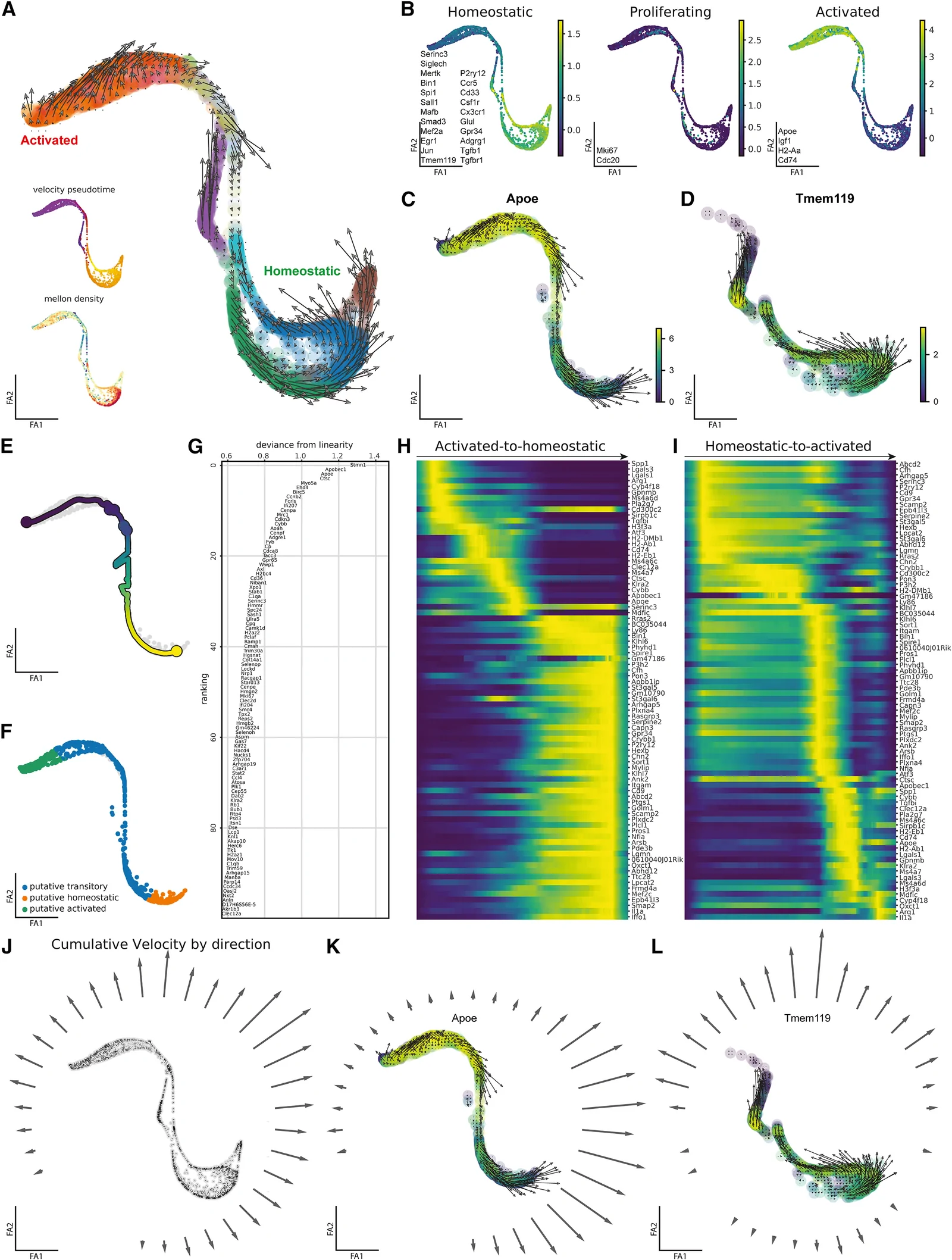
Unraveling the profiles of host microglia/macrophage continuous and reversible cell activation in response to transplantation (A) ForceAtlas2-based embedding with RNA velocity vectors, demonstrating the commitment of each cell toward the homeostatic or activated state. (B) Featureplots presenting the cumulative scores of homeostatic, proliferating, and activated programs for the microglia/macrophage states. (C) Activated-to-homeostatic subset of microglia/macrophage correlates with decreasing Apoe gradient along the activated-to-homeostatic trajectory. (D) Homeostatic-to-activated subset of microglia/macrophage correlates with decreasing Tmem119 gradient along the homeostatic-to-activated trajectory. (E) Activated-to-homeostatic subset of microglia/macrophage general trajectory reconstruction. (F) Activation states of microglia/macrophage population identified on the subset of activated-to-homeostatic microglia/macrophage. (G) Variable genes identified along the activated-to-homeostatic trajectory. (H) Variable genes identified upon RNA Velocity analysis on the activated-to-homeostatic subset, ranked by pseudotime. (I) Variable genes identified upon RNA Velocity analysis on the homeostatic-to-activated subset, ranked by pseudotime. (J) Cumulative velocity distance distribution for the complete dataset on the ForceAtlas2-based embedding. (K) Homeostatic-to-activated subset cumulative velocity distances, highlighting the major direction of commitment pointing to the top left side, i.e., the position of activated microglia/macrophage. (L) Activated-to-homeostatic subset cumulative velocity distances, highlighting the major direction of commitment pointing to the bottom right-right side, i.e., the position of homeostatic microglia/macrophage.

Using *Tmem119*^21^ and *Apoe*^14^ genes as markers of homeostatic and activated microglia, respectively, we show that activated-to-homeostatic transitioning (Figure 3C) correlates with a decreasing expression of *Apoe*, while the transitioning from homeostatic-to-activated (Figure 3D) demonstrates the trend of a decreasing expression of *Tmem119*. Activated-to-homeostatic subset was then used to build a common supervised trajectory starting from the most activated cell to the least activated, identifying three cell states i.e., “putative transitory”, “putative homeostatic” and “putative activated” (Figures 3E and 3F). The trajectory displayed with the gene IDs as a deviance from the healthy, with the genes located at the top of the trajectory defining the activated cell state, and the genes at the bottom defining the homeostatic cell state (Figure 3G).

In the same manner but for the RNA Velocity trajectory, with the idea of including potential local perturbations, we profile the activated-to-homeostatic trajectory (Figures 3C and 3H), and the homeostatic-to-activated (Figures 3D and 3I) trajectory with the variable genes along them. Transitioning from the activated-to-homeostatic (Figure 3H) was defined by the suppression of genes associated with activated microglia such as *Lgals3*, *Spp1*, *Cybb*, and *Apoe*, while transitioning from the homeostatic-to-activated state was defined by a suppression of a microglia homeostatic gene *P2ry12*, and at the same time upregulation of *Apoe*, a gene associated with microglia activation (Figure 3I).

For a quantitative assessment of cell commitment toward either the activated (preCS_a_) or the homeostatic (preCS_h_) state, and to profile the homeostasis balance between the two states, we introduced a numerical “*commitment score*” (CS) which summarizes RNA velocity vectors that follow a given direction either toward homeostatic or activated states. The cumulative velocity scores, representing the distribution of vectors for cell commitment for the homeostatic, and for the activated microglia, are shown in Figure 3J. They were further separated into two subsets: homeostatic-to-activated and activated-to-homeostatic, and for both trajectories, the preCS_a_ and preCS_h_ scores were quantified (Figures 3K and 3L).

The cumulative velocity/commitment score was used to summarize the directional balance of RNA velocity vectors between the activated and homeostatic poles. These panels quantify whether the myeloid population at the analyzed post-transplantation time point is preferentially directed toward activation or toward return to a homeostatic transcriptional state. This provides a quantitative complement to the trajectory and marker-expression analyses and supports our interpretation that host myeloid activation after transplantation is dynamic and at least partially reversible.

We explore the state commitment score of microglia/macrophage population (CS) as a ratio of the homeostatic and activated commitment scores. The value of unnormalized commitment score (unCS) = 9.335. We normalize this value by assessing the number of cells within each state. The value of normalized commitment score (nCS) = 8.066, meaning the general trend of the population commitment is highly shifted toward the homeostatic state. The commitment of cells toward the homeostatic state is between 8.066 and 9.335 times higher compared to the commitment toward the activated state.

### Differential expression of selected genes in the host mouse retina following xenotransplantation of human RGCs

The expression of genes identified to be variable across the homeostatic-to-activated and activated-to-homeostatic fates was validated by immunohistochemistry. Retinal whole mounts were stained for Spp1, Tmem119 and Apoe three days after RGC transplantation (Figures 4A–4K and 4O–4U). Close to the injection site, we noted a localized increase in density, and a change in morphology of the host GFP+ myeloid cells.

**Figure 4:**
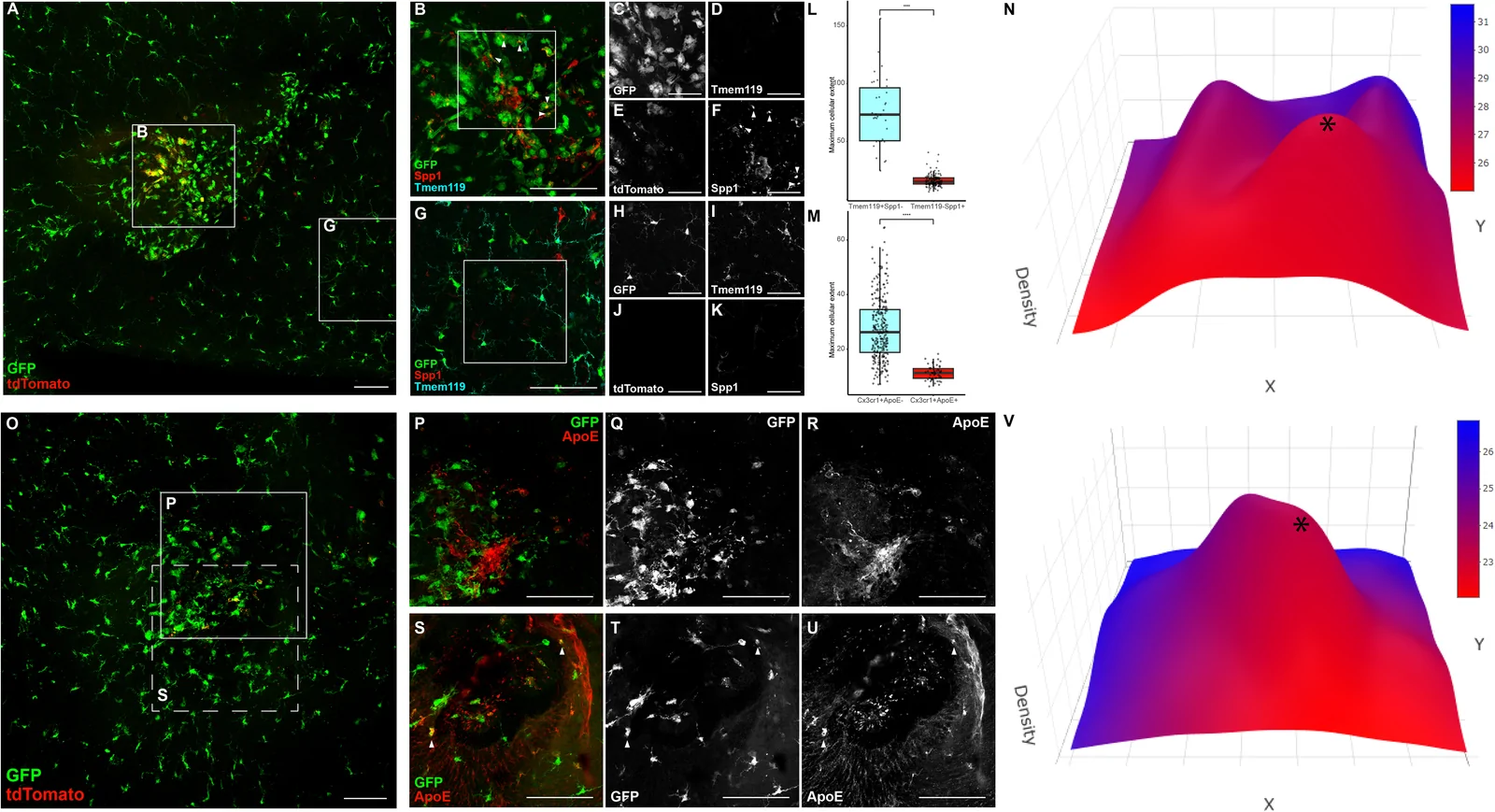
Injection of tdTomato^+^ hESC-derived RGC results in a localized increase in density of Cx3cr1-GFP^+^ myeloid cells (microglia and macrophages), and loss of characteristic homeostatic microglia traits including ramified morphology and expression of the marker Tmem119 (A) Injection sites, identified by tdTomato (A and E), display highly localized increases in density of GFP-labeled myeloid cells (B), along with transition to amoeboid morphology (B and C), in contrast to nearby regions of the retina (G and H). Likewise, expression of homeostatic microglial marker Tmem119 is absent within the injection site (D) but persists in ramified microglia elsewhere in the retina (I), whereas the DAM marker Spp1 is found in GFP+ myeloid cells at the injection site (B and F) but not those elsewhere (G and K). Additional immunohistochemical investigation (O–U), both at the surface (P–R) and within the retina (S – U) reveals localized increase in Apoe expression at injection sites, including in amoeboid GFP+ myeloid cells below the retinal surface (M–Q). Scale bars = 100 μm (A, B, G, and O–U), 50 μm (C–F, H–K). (L and M) Bar charts representing maximum cellular extent for Tmem119+Spp1- (34 cells) vs. Tmem119-Spp1+ (78 cells) (L) and Cx3cr1+ApoE- (159 cells) vs. Cx3cr1+ApoE+ (38 cells) (M). Asterisk highlights significance *p* < 2.2e−16. Cells includes in the measurements are the ones visible on the panels (A and O). (N and V) Density plots demonstrating distribution of microglia cells for both Tmem119+ and Spp1+ (N), Apoe- and Apoe+ (V), colored by binned maximum cellular extent. Injection site is marked with asterisk (∗). n of experimental repeats = 1.

The spatial distribution observed by immunohistochemistry is consistent with the heterogeneity detected by scRNA-seq. Because GFP-positive myeloid cells were purified from whole transplanted retinas, the sequenced population likely includes activated microglia/macrophages concentrated near the injection site as well as more homeostatic microglia from distal retinal regions. The transcriptional continuum from homeostatic to activated states observed in the single-cell analysis reflects, at least in part, the spatially heterogeneous exposure of host myeloid cells to the transplanted donor RGCs and local injury signals. The host myeloid cells were noted to be less ramified and more ameboid in shape compared to the same cell type further away from the injection site. Expression of the homeostatic microglial marker Tmem119 was absent at the injection site but was present in the ramified microglia elsewhere in the tissue. The DAM marker Spp1 was expressed in GFP+ myeloid cells at the injection site (B, F) but not elsewhere (G, K) (of note, Spp1 is also expressed by a subpopulation of retinal ganglion cells). Additional immunohistochemical investigation (O- U), both at the surface (P- R) and within the retina (S- U) reveled localized increase in Apoe expression at the injection site. These findings confirm that host myeloid cells in proximity of transplanted RGC change their morphology and upregulate classic DAM markers Spp1 and Apoe.

Maximum cellular extent (the longest linear distance across a cell, measured from the distal end of one process to the distal end of the opposite process through the cell body) quantification results demonstrate significantly higher cellular extent for Tmem119+Spp1- vs. Tmem119-Spp1+ microglia (Figure 4L), and similar pattern is observed for Cx3cr1+ApoE- vs. Cx3cr1+ApoE + microglia (Figure 4M), confirming Tmem119 as a marker of homeostatic microglia, and Spp1 and ApoE as markers of activated microglia. *p* value for both quantifications is <2.2e−16.

Separately, we built 3D density plots to compare microglia distribution and maximum extent length (i.e., phenotype) within and outside the injection site (Figures 4N and 4V). The injection site is marked with asterisk (∗). The observed density peaks correlate with the injection site, highlighting microglia accumulation. Similarly, maximum extent length is observed to be lower at the injection sites, suggesting more of activated rather than homeostatic microglia accumulation within the injection area.

*In silico* integration of myeloid/lymphoid cell populations from the developing, adult, and damaged mouse retina reveals novel mechanisms of host response to RGC transplantation

To determine whether the host myeloid response to RGC transplantation resembles myeloid activation programs observed in other retinal stress and disease contexts, we designed an *in silico* experiment based on single-cell RNA-sequencing public data integrated analysis. We integrated myeloid cell populations (Figure 5A) from the following conditions:

(a) Developing retina available here: https://cellxgene.cziscience.com/e/7d69613a-9e75-4bda-8a9f-9088073bdd1c.cxg/.
(b) Healthy adult retina available here: https://cellxgene.cziscience.com/e/3375d9f3-e17b-46da-b40e-ec6e1779ad88.cxg/.
(c) Glaucoma model (microbead-induced IOP elevation) available here: https://cellxgene.cziscience.com/e/a60147e0-5a01-4b30-bbc7-13c8ff9cf9eb.cxg/.
(d) Optic nerve crush model available here: https://cellxgene.cziscience.com/e/46c7eca0-7db1-411e-baf3-6f7334140422.cxg/.
(e) Myeloid cell population from the host retina after transplantation of human RGC (original data).

**Figure 5:**
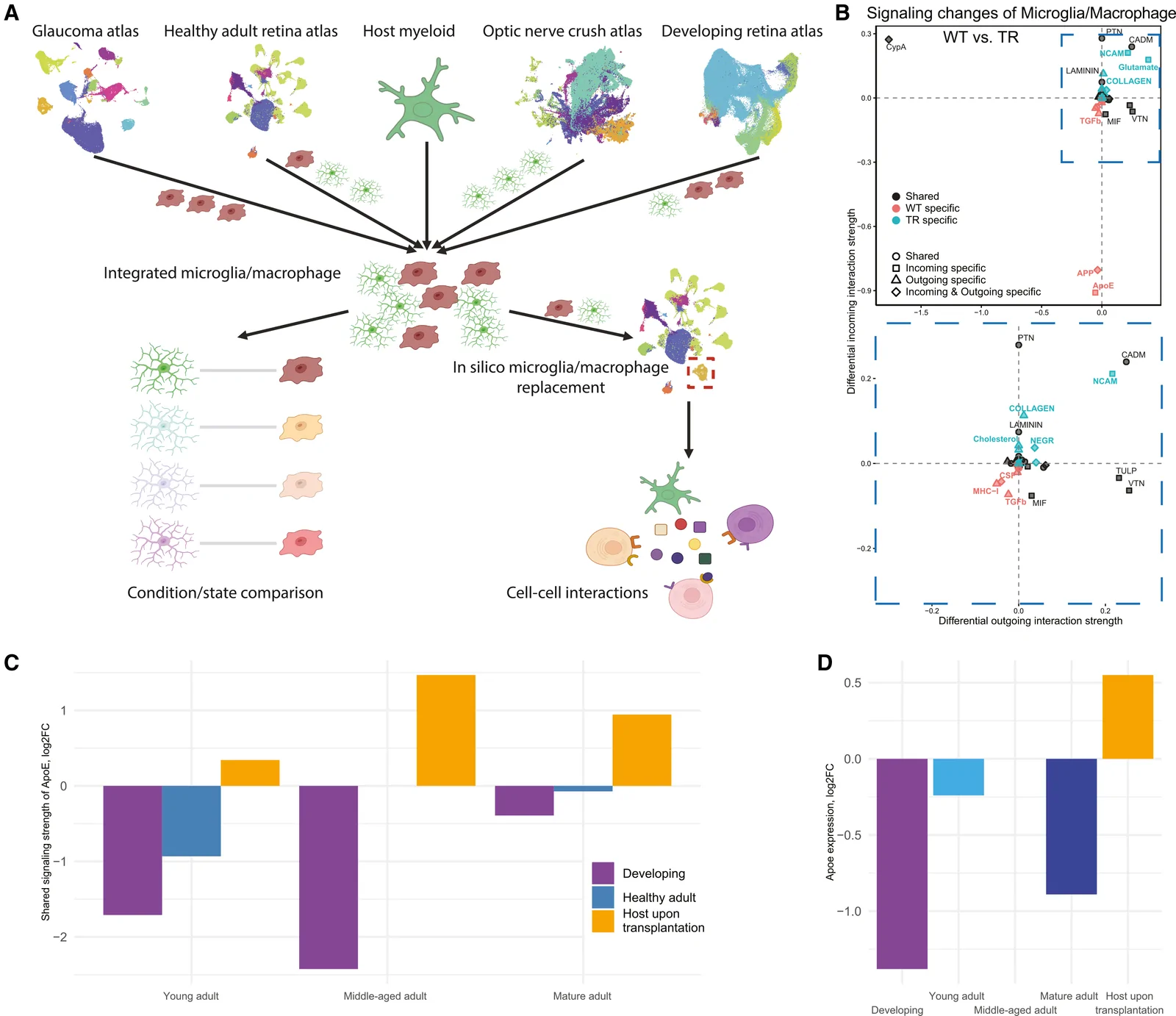
*In silico* integration of the developing, adult, and damage conditions myeloid population reveals the mechanism of host response to transplantation (A) Abstract sketch demonstrating the *in silico* pipeline for the integrated microglia/macrophage downstream analysis (green ramified cells are the illustrations of homeostatic microglia, and red ameboid cells illustrate activated microglia). (B) Cell-cell interactions comparison of healthy adult mouse retina microglia/macrophage versus host upon transplantation microglia/macrophage in the 20 weeks old healthy adult mouse retina surrounding. The interactions cover signaling coming to Microglia/Macrophage from all the other populations (incoming), and signaling coming from Microglia/Macrophage to all other populations (outgoing). WT = healthy adult retina, wild-type, TR = host-upon-transplantation. (C) Plot representing summarized incoming and outgoing signaling strength of Apoe signaling in developing, healthy adult, and host upon transplantation microglia/macrophage in the surrounding of young adult, mature adult, and aged retinal cell classes of mouse healthy adult retina atlas. (D) Apoe expression in microglia/macrophage of developing, young adult, mature adult, aged, and host upon transplantation conditions.

The integrated dataset of microglia/macrophage presents 2,814 cells in host-upon transplantation, 2,306 cells in developing, 10,597 cells in healthy adult, 5,353 cells in glaucoma model, 17,464 cells in optic nerve crush, total spanning 38,534 cells.

*In silico* myeloid replacement experiments were performed by computationally substituting the myeloid population from one condition into the retinal cellular context of another condition. For example, the native myeloid population, originally present in the healthy adult retina atlas was, removed and replaced with myeloid cells from the optic nerve crush, glaucoma, developing retina, or post-transplantation condition. In this reconstructed dataset, the overall retinal cell-class structure was preserved, while the exchanged myeloid compartment retained the transcriptomic profile of its original condition. CellChat was then applied to the reconstructed datasets to compare predicted ligand-receptor communication between condition-specific myeloid cells and the shared retinal cellular environment. This *in silico* experimental approach allows us to create a unique biological model for exploring cell-cell interactions between different conditions.

For the first part of *in silico* experiments, we used the “20-week time point” dataset to mimic cells from an adult mouse host retina, one into which RGC would typically be transplanted. We compared signaling cascade of myeloid cells within the native healthy adult 20-week time point, between the native myeloid cell population and the myeloid cells from the host. Cell-cell interactions were grouped into two categories: incoming for signals where microglia/macrophage is receiving, and outgoing where microglia/macrophage is sending the signals to/from other cells, respectively. Cell-cell interactions analysis revealed several unique signaling mechanisms including increased signaling strength in NCAM, glutamate, and COLLAGEN, and decreased signaling strength of TGFb, Apoe, and APP (Figure 5B).

Next, we profiled cell-cell interactions in three additional groups including:

• Young adult (≤14 weeks old).
• Mature adult (15 weeks–21 weeks old).
• Aged adult (>35 to 43 weeks and 52 weeks old).

And used three sets of myeloid cell populations to populate the healthy adult atlas i.e.,

(1) Native healthy adult myeloid.
(2) Developing (E10-P8),
(3) Host myeloid from the retina upon human RGC transplantation.

Apoe was found to be differentially expressed in microglia/myeloid across the studied conditions.

Apoe was downregulated in development, in the young adult, and in the healthy aged animals, and upregulated in the host upon RGC transplantation (Figures 5C and 5D). This Apoe differential expression pattern is in line with reported expression of Apoe in DAMs.^10^ We observed the lowest Apoe level in the developing myeloid cells and the highest in host-upon-transplantation condition. We also observe continuous increase with aging win Apoe expression increasing in young adult, then aged adult, and highest in the mature adult. These findings indicate that the developing and young adult retina may represent less inflammatory host environments and therefore generate the testable hypothesis that developmental or young adult retinal contexts could be more permissive for transplantation, as retinal cells at this age showed the least activation response to transplantation. With the observed Apoe expression pattern across the different conditions, we then correlated Apoe signaling strength in the myeloid cell population with the Apoe expression between the conditions of developing, healthy adult (young, mature, and aged), and host upon transplantation myeloid populations (Figure S4). Interestingly, similar trend was observed with the highest Apoe signaling strength for host upon transplantation condition, at every studied time point

### Myeloid cell populations across the different conditions have a differential microglia activation profile

To evaluate microglia activation, inflammatory response, and other pathways, we perform single-sample gene set enrichment analysis (ssGSEA) pathway analysis from the following conditions of the myeloid population: developing (E10-P8), healthy adult (4–52 weeks), host upon transplantation (6 months), optic nerve crush ≤ 4 days (12 h, 1 day, 2 days, 3 days, 4 days), optic nerve crush >4 days (7 days, 14 days), and microbead glaucoma model (7–22 weeks) (Figure 6A). The analysis focused on inflammatory pathways and showed that microglia/macrophages from the developmental cell state had the lowest normalized enrichment score (NES) in each of the analyzed pathways. The highest NES was observed in the host upon transplantation condition for most pathways.

**Figure 6:**
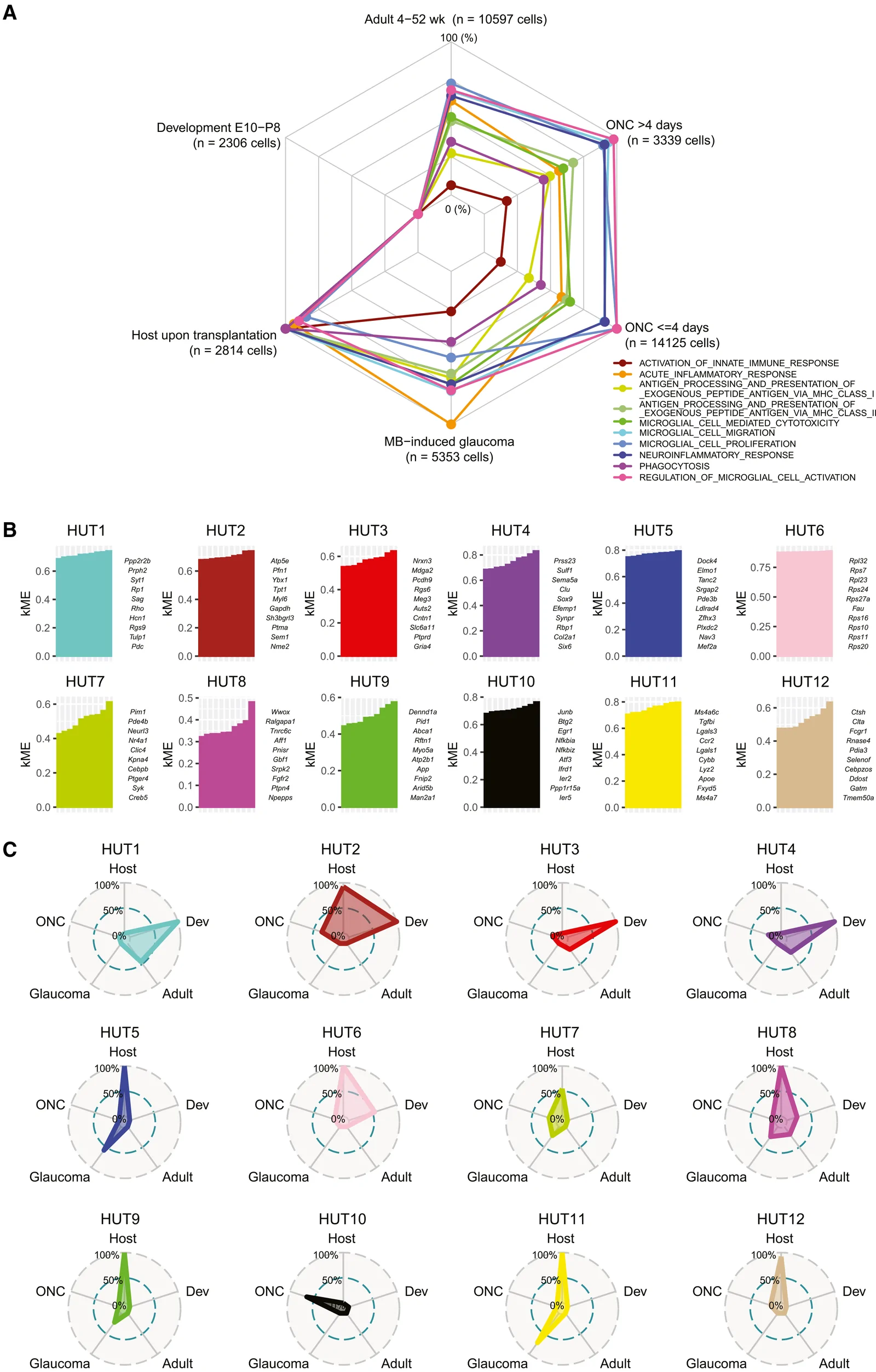
Integrated myeloid dataset comparison demonstrates differences in microglia activation (A) Polygon chart representing the results of ssGSEA pathways analysis on inflammatory-oriented pathways in the developing, healthy adult, optic nerve crush, glaucoma model, and host upon transplantation microglia/macrophage conditions. (B) WGCNA analysis represented in 12 modules with top 10 hub genes in each module. WGCNA modules are labeled by their unsupervised module identifiers. Condition associations indicate the condition in which each module shows relatively higher module score/eigengene enrichment and should not be interpreted as exclusive condition identity. (C) Polygon chart demonstrating the modules distribution across the conditions from the integrated myeloid dataset.

Of the pathways analyzed, GOBP_ACUTE_INFLAMMATORY_RESPONSE had the highest NES in the glaucoma condition, while GOBP_REGULATION_OF_MICROGLIAL_CELL_ACTIVATION along with GOBP_MICROGLIAL_CELL_PROLIFERATION had the highest NES for the ONC condition. This enrichment indicates a similarity in microglia activation pattern between disease conditions and host cell response to RGC transplantation (Figure 6A).

A closer look at the genes involved in the enriched pathways indicated a differential expression pattern. In GOBP_ACTIVATION_OF_IMMUNE_CELLS pathway, several genes were differentially upregulated in the host including: Sting1, Arrb2, Nlrp6, Nfkbil1, Mark4, Akt1, Myd88, TLr8, and Sec1411 Figure S8. These genes were uniquely upregulated in the host retina post transplantation but were suppressed in the MB-induced glaucoma model, in the ONC model, and during development. In this manner, we provide the complete list of the genes along with their relative expression involved in the pathway analysis between the conditions, in Figure S8.

Using the same dataset we performed whole genome correlation network analysis (WGCNA) in an unsupervised manner. The analysis revealed twelve HUT modules (Figures 6B, 6C, S5A, and S7), with HUT1 being specific for the healthy (developing and adult), HUT3 and HUT4 for developing, HUT6, HUT8, HUT9, and HUT12 for host upon transplantation, HUT5 and HUT10 for optic nerve crush condition, and HUT11 for host upon transplantation and MB-induced glaucoma.

HUT11 represents an activation module, with several of the hub genes including *Ccr2*, a receptor for *Ccl2*, a chemoattractant important for macrophage and monocyte recruitment, *Lgals1*, *Cybb*, *Lyz2*, and *Apoe*, genes associated with microglia activation in disease (Figures 6C and S6). This module was significantly expressed in host upon transplantation and glaucoma conditions (Figure S5). The optic nerve crush condition was represented by HUT10 and it includes genes as *Junb*, *Egr1*, and *Atf3*, known transcription factors that modulate activity of several other genes. The healthy condition (developing and adult) was characterized by HUT1, which included several genes including *Rp1*, *Sag*, *Rho*, *Tulp1*, and *Pdc*, which genes are implicated retinal developmental diseases such as retinitis pigmentosa. From the selected the modules with the highest score in host upon transplantation condition (Figure 6C) we build the hub gene network (Figure S5B), where HUT2, HUT6, and HUT11 form a distinct cluster, and genes from HUT8 and HUT9 form another cluster. On the other hand, HUT10, specific to ONC, represents a set of genes known to optic nerve crush and participating in optic nerve injury, cell death, and survival (*Junb*,^22^ immediate-early response genes *Egr1*, *Atf3*,^23^ *Nfkb*,^24^ *Ier2*,^25^^,^^26^ *Ifrd1*, and^26^ *Ppp1r15a*^27^).

## Discussion

The success of RGC restoration by transplantation depends on several factors including the state of the host microenvironment.^28^^,^^29^ In this study, we aimed to understand host myeloid cell response following the transplantation of human stem-cell derived RGCs. We utilized a combination of single-cell RNA sequencing, advanced integrated transcriptomic analysis, and immunohistochemistry to characterize the host myeloid cell reactivity to RGC transplantation.

### Large-scale single-cell atlases integration and its application

Single-cell datasets of the mouse retina, including comprehensive adult retina atlas by Li et al.,^30^ have been integral for analyzing transcriptional signatures of cell classes and types. However, they are not representative of rare cell types, such as microglia and are limited to healthy state only.^31^^,^^32^ For a comprehensive understanding of development and disease a large-scale atlas integration approach is necessary. In the present study we assembled such atlas as complementary, study-specific integrated reference built from multiple publicly available datasets. This allowed us to harmonize adult mouse retinal transcriptomes across studies and use the resulting atlas for reference-based annotation of our transplantation dataset, with a focus on myeloid populations. Compared with previously published resources, our atlas incorporates a broader collection of public datasets and contains 373,651 cells, thereby increasing representation across studies, ages, disease models, and experimental contexts. This integrated structure was essential for our downstream analyses comparing host myeloid cells after RGC transplantation with healthy adult, developing, glaucoma-associated, and optic nerve crush-associated myeloid states. This atlas can be used as a control dataset for multi conditional studies, and/or serve as a reference tool when analyzing other single-cell RNA-seq datasets e.g., for automated cell annotation. One should caution when using such Atlas: it allows to study the gene expression and trajectory reconstruction when built-in controls are used - mitochondrial, ribosomal, or MALAT1.^33^ However, such an atlas should not be used to analyze frequency of populations or their aging.^34^^,^^35^ This atlas could also serve as a control, “natural history” group without the requirement of collecting the control samples in an independent experiment.

This mouse control adult retina atlas, spanning 4 to 52 weeks of age allows to study the maturation and aging of individual cell classes. We demonstrated a continuity of timepoints in the integrated atlas, and this can enable cell class-oriented studies: for instance, a user may subset the amacrine cell class and explore the transcriptional changes occurring over time. The metadata such as sex, sequencing depth and platform, and multiple other variables, are important for both biologically and technically oriented studies. One example is a sample variance analysis,^34^ which can be now be performed within the atlas.

Establishment of the reference atlas was then followed by annotating our single-cell RNA seq data, with reference to the atlas. For this study, we use the terminology “myeloid” to describe all possible myeloid cell populations collected from the retina before and after donor RGC transplantation, and these include Cx3cr1+ cells (microglia, resident macrophages, monocyte-derived macrophages), Cx3cr1^low^ activated cells with an activated transcriptional profile, and other myeloid cells. Due to the complexity of microglia and macrophage profiling, we use the term “microglia/macrophage” to highlight the population of our interest in the analysis. Since this study is function-oriented and activation-oriented, we did not perform further separation to microglia and macrophages when it comes to cell fate trajectory inference or any other downstream analysis.

### Cell fate trajectory features of host microglia

Upon cell trajectory analysis we confirm an existing paradigm that microglia/macrophage population of the retina is a dynamic system, and each cell within this system may exist in either the “homeostatic”, “activated”, or the “transitory” state. We also profile the continuity and bidirectionality of the changes. These confirmed features align with the previous discoveries performed on microglia in healthy wild type mouse retina and the retina of microbead-induced glaucoma mouse model,^36^ proposing the activation mechanisms to be flexible, reversible and bi-directional. Previously demonstrated cell fate trajectory reconstruction methods highlight the system to be controllable, and the homeostatic-activated homeostasis to be a unique condition-related feature. The application of cell fate trajectory inference methods^37^ assumes that we are working with the dynamic system. Here, we applied RNA velocity method to reconstruct the evolution of host microglia/macrophage population with the dynamics in the activation profile in response to RGC transplantation. The myeloid population of the healthy adult retina can also be analyzed with similar strategy due to the ongoing myeloid cell renewal throughout life. This creates necessary dynamic, that permits potency-based deconvolution and allows to apply scFates algorithm.

We observe a well-defined transitory state of microglia/macrophages between the homeostatic and activated tips. Our analysis suggests the presence of two transitory populations, representing the directionality of these changes: homeostatic-to-activated and activated-to-homeostatic. While these two populations are very similar in their transcriptional profile, the Euclidian distance suggests that these transitory programs are different and are not the same program executed in the opposite direction. In our experimental design with the focus on microglia/macrophage activation, we expect the bi-directional nature of changes. However, we appreciate the several discovered populations that include pro-angiogenic and IFN-response microglia/macrophages. Yet, we observe these populations to be blended with the rest of the microglia/macrophage population, suggesting that microglia/macrophages potential to activation is universal and function-agnostic. Further, high-resolution studies could provide an answer to the question of bi-directional cell state transition.

### Commitment score

In this manuscript, we also introduce a concept of the “commitment score”. For the microglia/macrophage population it is calculated based on the two-directional commitment model: the cells studied can go either to the activated or to the homeostatic state. However, this paradigm can be expanded further to quantitatively describe multi-directional systems. For example, mouse and human PAX6+ retinal progenitors give rise to amacrine, horizontal, and RGC.^38^^,^^39^^,^^40^ Other developing systems including neural crest, brain, PBMC may contain three and more final cell states arising from the same progenitor state. In this case, a solution would be to build the force-directed layout, such as ForceAtlas2, or applying the circular projection with the number of vertices equal to the number of final states^41^^,^^42^ With that, each sector of the plot will have equal weight, when quantifying the cumulative velocity distance and measuring the commitment score.

The decision to use unnormalized or nCS should be based on an experimental question and design. Since the main difference between these two metrics is in the cell number of adjustment, it is important to know the reason of such cell number variation. This could be the true condition-related frequency difference, sample variation, or the library preparation-related issue. Unless it is possible to determine the reasoning, the true commitment score value lays between the unnormalized and normalized, where stronger condition-related frequency change is a hint to look closer to the nCS value, and stronger batch-related frequency change can be more associated with the unnormalized value. In the real case scenario, additional methods to identify the reasoning of frequency change, such as Bayesian models application for the dataset composition analysis, could help in finding out the real commitment score.

### Microglia states

Through unsupervised clustering not based on known microglia-specific genes, wconfirm previous observation that *Tmem119* and *P2ry12* are markers of homeostatic microglia.^43^^,^^44^ The activated state was characterized by upregulation of *Apoe*, *Spp1*, *Lgals3*, *Lgals1*, *Cybb*, *Atf3*, *Cd74*, *Il1a*, and *Apobec1* and downregulation of *Tmem119* and *P2ry12*. Interestingly, this activated expression pattern is highly similar to that of DAM, also known as neurodegeneration-associated microglia (MGnD) which were first described in the context of Alzheimer’s disease, multiple sclerosis, and amyotrophic lateral sclerosis.^19^^,^^45^ The same transcriptional signature was subsequently described in the retina in glaucoma and photoreceptor degeneration mouse models, as well as human glaucoma and age-related macular degeneration retinal tissues.^14^^,^^46^ Interestingly, these prior studies have demonstrated that the DAM/MGnD signature can be induced by microglial phagocytosis of apoptotic or damaged neurons *in vivo* in the brain and in the retina.^19^ Furthermore, the same DAM-like signature has been identified in the developing murine retina, where it is thought to result from the developmentally appropriate microglial elimination of excess apoptotic RGC.^47^^,^^48^^,^^49^^,^^50^ Thus, it seems likely that the DAM-like signature seen following human RGC transplantation results from retinal microglia phagocytosing transplanted RGC, and that modulating this myeloid cell response could enhance transplanted RGC survival.

### Cell-cell interaction

Cell-cell interaction analysis with CellChat, applied in the manuscript, should be performed carefully, when applying it on the multibatch data. Due to the different composition of datasets (batches), it is important to adjust for the population presence and its size. In our case, we did not include the time points that were missing any of the major retinal classes, since it affects the general pool of ligands and receptors and may pull the picture to the false results. We have also excluded the time points with low abundance of microglia/macrophage cells. Nevertheless, even these filters do not promise the real cell-cell interactions picture. Particular timepoints may be outlying due to the different platforms, sequencing depth or coverage in the study. In this case, averaging or binning the data may be a solution to override the noise.

### Multiconditional analysis of microglia

The GSEA pathway and WGCNA analyses allowed us to quantitatively compare several signaling pathways related to inflammation and their enrichment scores across multiple conditions. Our findings suggest that human RGC transplantation is a stronger stress factor compared to optic nerve crush or microbead-induced glaucoma and is the strongest across all conditions. Upon transplantation, microglia/macrophages upregulate MHC-I and MHC-II receptors. Their acute inflammatory response is very similar as in glaucoma model condition, yet different compared to optic nerve crush model. Also, transplantation is the only condition among those studied that resulted in microglia/macrophages to extremely increase phagocytosis function. However, we also confirm microglia activation in optic nerve crush and glaucoma model conditions that could be one of the mechanisms of RGC death. In contrast, the behavior of developing microglia/macrophages is completely different from any other condition studied. Unlike healthy adult, optic nerve crush model, glaucoma model, or post-transplantation condition, developing microglia/macrophages have the lowest inflammatory potential.

We included datasets of the developing retina to explore further the known phenomena that the immature retinal environments have been described as more permissive for donor neuron survival and integration.^51^^,^^52^^,^^53^^,^^54^^,^^55^^,^^56^^,^^57^^,^^58^^,^^59^ This comparison is directly relevant to the central question of whether host myeloid cells after RGC transplantation resemble homeostatic adult retina, injury/disease-associated retina, or developmental retinal states. Our analysis suggests that the developing retina is the most accepting host environment for donor neurons in the context of host microglia activation. Thus, for multiple inherited disease, including retinitis pigmentosa, Leber congenital amaurosis (LCA), Stargardt disease, and cone-rod dystrophies, transplantation into the developing system, could be a promising solution in absence of other therapy. Additionally, transplantation in the developing system settings allows further fundamental research: while the settings allow longer lifespan of donor neurites, they may reveal their full functional potential, if any, without the risk of being arrested due to the transplantation/neurogenesis in regeneration-unfriendly host environment.^4^^,^^52^^,^^53^^,^^60^^,^^61^^,^^62^^,^^63^^,^^64^

Overall, our work underlines the importance of modulating host innate immunity, including microglia and macrophages, to improve neuron transplantation into the retina. The advanced integrated transcriptomics analysis showed that retinal microglia activation in response to neuron transplantation is profound and more dramatic than microglial response in RGC damage models. However, we also demonstrate the reversibility of this process using the pseudotime analysis, suggesting a molecular roadmap to decrease the activation of host myeloid population and increase the return to the homeostatic state.

### Limitations of the study

1. Because the single-cell dataset was collected at a single acute time point, three days after transplantation, the transcriptionally homeostatic population cannot be unambiguously separated into cells that were never activated and cells that had previously been activated and returned to a homeostatic profile. Although RNA velocity supports bidirectional movement between activated and homeostatic states, definitive resolution of these cellular histories would require additional time points or lineage-tracing approaches.
2. The present study focuses on the acute host myeloid response at day 3 after transplantation and does not resolve the earliest molecular events occurring during the first 24 h after donor-cell delivery. Earlier time points, including day 1 post-transplantation, would likely provide additional insight into the initiation of antigen processing/presentation, phagocytic signaling, and donor-cell loss. Similarly, a longitudinal quantification of donor-cell survival across multiple early time points would be required to define the kinetics of graft clearance. These experiments represent an important direction for future studies but were outside the scope of the current work.
3. Because a matched sham/vehicle-injected parallel scRNA-seq control was not included, the present study cannot fully distinguish transcriptional programs induced specifically by donor RGCs from those induced by injection-related local perturbation. Prior retinal studies have shown that intraocular injection itself can activate retinal microglia and inflammatory signaling,^65^ and sham-injection controls have been used in subretinal and intravitreal cell-transplantation studies to account for injection-associated effects.^66^^,^^67^^,^^68^ To mitigate this limitation, we compared the host-upon-transplantation myeloid population to multiple publicly available retinal myeloid states, including healthy adult retina, developing retina, optic nerve crush, and microbead-induced glaucoma.
4. The present study does not focus on sex-associated changes. Thus, while the metadata is provided, we did not perform any experiments on studying association of sex and results.

## Resource availability

### Lead contact

Requests for further information and resources should be directed to and will be fulfilled by the lead contact, Petr Baranov (peb112@pitt.edu).

### Materials availability

This study did not generate new unique reagents.

### Data and code availability

• The host upon transplantation single-cell RNA sequencing data generated in this study is deposited at GEO and available under the GSE285564 reference number and are publicly available as of the date of publication.
• Mouse healthy adult retina atlas is available on CELLxGENE: https://cellxgene.cziscience.com/collections/7dd7e8a1-f7c2-4213-9f23-dd52afb7ee16. Researchers interested in contributing data for future versions of the reference atlases should contact the corresponding authors directly.
• This paper analyzes existing, publicly available data,^69^^,^^70^^,^^71^^,^^72^^,^^73^^,^^74^^,^^75^^,^^76^^,^^77^^,^^78^^,^^79^^,^^80^^,^^81^^,^^82^^,^^83^^,^^84^^,^^85^^,^^86^^,^^87^^,^^88^^,^^89^^,^^90^^,^^91^^,^^92^^,^^93^^,^^94^^,^^95^^,^^96^ referenced and described in Table S1. We do not claim any authorship over the original datasets provided in Table S1 and labeled as not published at the moment of the manuscript submission.
• All original code has been deposited at GitHub and is publicly available at https://github.com/mcrewcow/neurons_transplantation_microglia as of the date of publication.
• Any additional information required to reanalyze the data reported in this paper is available from the lead contact upon request.

## Acknowledgments

P.B. Department of Defense VRP FTTSA VR220053, 10.13039/100016618Gilbert Family Foundation, MGB GCTI Award. A.M. 10.13039/100002924The Glaucoma Foundation (TFG) and 10.13039/100001818Research to Prevent Blindness (10.13039/100001818RPB) Glaucoma Fellowship, sponsored by Patricia Hill. P.F.C. NIH/NEI 2T32EY007145 M.A.M. NIH/NEI R01EY035312, the 10.13039/100001641Glaucoma Research Foundation Catalyst for a Cure Award, the 10.13039/100015112Melza M. and Frank Theodore Barr Foundation, the Robert M. Sinskey, MD, Foundation, the Ruettgers Family Charitable Foundation, and the B.L. Manger Foundation. D.F.C. Department of Defense HT9425-23-1-1045, NIH/NEI R01EY031696, and P30 EY003790 core grant.

## Author contributions

E.K.; bioinformatics analysis, experimental design and lead first author. A.M.; tissue dissociation, single-cell suspension preparation, cell sorting and sorted population characterization; library preparation and logistics for sequencing. sample processing and second first author. N.R.; cell differentiation; V.V.M.; RGC transplantation and tissue dissection, histology. E.K., S.V., N.B., and E.L.collection, parsing, and processing of public single-cell RNA sequencing datasets; P.F.C. and G.B. immunohistochemistry and imaging; H.T.K. and E.K. microglia image analysis and visualization; M.A.M. and P.B. conceived the study. E.K., A.M., M.A.M., D.F.C., and P.B. wrote the manuscript. All authors approved the final manuscript.

## Declaration of interests

P.B. laboratory, none; M.A.M. laboratory, none; D.F.C. laboratory, none.

## STAR★Methods

### Key resources table

**Table:** 

REAGENT or RESOURCE	SOURCE	IDENTIFIER
**Antibodies**		

Guinea pig anti-Tmem119 primary antibody	Synaptic Systems	Cat#400 004; dilution 1:500
Goat anti-Spp1/osteopontin primary antibody	R&D Systems	Cat#AF808; dilution 1:500
Guinea pig anti-Apoe primary antibody	Synaptic Systems	Cat#465 004; dilution 1:500
Donkey anti-guinea pig 405 secondary antibody	Jackson ImmunoResearch	Cat#706–475-148
Donkey anti-guinea pig 647 secondary antibody	Jackson ImmunoResearch	Cat#706–605-148
Donkey anti-goat 647 secondary antibody	Invitrogen	Cat#A32849

**Biological samples**		

Mouse retinas from CX3CR1^GFP^ knock-in mice after subretinal transplantation	This paper	N/A

**Chemicals, peptides, and recombinant proteins**		

Matrigel	Corning	356254
mTeSR1 medium	STEMCELL Technologies	85857
Accutase	Thermo Fisher Scientific	A1110501
Y-27632 ROCK inhibitor	LC Laboratories	Y-5301
IWR-1-endo	Selleckchem	S7086
BMP4	PeproTech	120-05ET
Smoothened agonist (SAG)	Fisher Scientific	50-002-62807
Taurine	Sigma Aldrich	T0625-100G
All-trans-retinoic acid (ATRA)	BioGems International	3027949
DAPT	Fisher Scientific	26-341-0
BDNF-loaded PODS	Cell Guidance Systems	PPH1-250
GDNF-loaded PODS	Cell Guidance Systems	PPH2-250
ProLong Diamond Antifade Mountant	Thermo Fisher Scientific	P36970

**Critical commercial assays**		

Chromium Next GEM Single Cell 3 prime Reagent Kits v3.1	10× Genomics	CG000204
Embryoid Body Dissociation Kit	Miltenyi Biotec	130-096-348
Adult Brain Dissociation Kit, mouse and rat	Miltenyi Biotec	130-107-677
CD90.2 MicroBeads, mouse	Miltenyi Biotec	130-121-278

**Deposited data**		

Original study raw and processed single-cell RNA-seq data from host retinal myeloid cells after transplantation	This study	GEO accession: GSE285564
Healthy adult mouse retina atlas	This study/CELLxGENE	https://cellxgene.cziscience.com/e/3375d9f3-e17b-46da-b40e-ec6e1779ad88.cxg/
Other assembled atlases (developing, optic nerve crush, laser-CNV, glaucoma retina atlases); Analysis-ready integrated myeloid objects	This study/CELLxGENE	https://cellxgene.cziscience.com/e/3375d9f3-e17b-46da-b40e-ec6e1779ad88.cxg/
Public single-cell RNA-sequencing data	GSE accessions and DOIs listed in Table S1 https://cellxgene.cziscience.com/collections/7dd7e8a1-f7c2-4213-9f23-dd52afb7ee16	GSE accessions and DOIs listed in Table S1 https://cellxgene.cziscience.com/collections/7dd7e8a1-f7c2-4213-9f23-dd52afb7ee16

**Experimental models: Cell lines**		

Human: H9-BRN3B:tdTomatoThy1.2 hESC reporter line	This paper/author laboratory: provided by Dr. Donald Zack	Source of parental H9 line: H9 (WiCell, Madison, WI, https://www.wicell.org); Identifier/RRID: CVCL_9773; Sex: Female; mycoplasma status: negative tested

**Experimental models: Organisms/strains**		

Mouse: CX3CR1^GFP^ knock-in mice on C57BL/6 background	The Jackson Laboratory	Strain #:005582; RRID:IMSR_JAX:005582 (https://www.jax.org/strain/005582)

**Software and algorithms**		

Cell Ranger v7.0.1	10× Genomics	https://support.10xgenomics.com/single-cell-gene-expression/software
Mouse reference genome 2024-A Mouse GRCm39	10× Genomics	2024-A Mouse GRCm39
Seurat v4.3.0.1	Satija Lab^97^	https://satijalab.org/seurat/
DoubletFinder	McGinnis et al.^98^	https://github.com/chris-mcginnis-ucsf/DoubletFinder
velocyto	La Manno et al.^99^	https://github.com/velocyto-team/velocyto.py
samtools	Li et al.^100^	https://github.com/samtools/samtools
scVelo	Bergen et al.^101^	https://github.com/theislab/scvelo
Harmony	Korsunsky et al.^102^	https://github.com/immunogenomics/harmony
scVI	Lopez et al.^103^	https://github.com/scverse/scvi-tools
scFates	Faure et al.^104^	https://github.com/LouisFaure/scFates
Azimuth	Satija Lab^97^	https://github.com/satijalab/azimuth
CellChat v2.1.0	Jin et al.^105^^,^^106^	https://github.com/jinworks/CellChat
escape v1.10.0	Borcherding et al.^107^	https://bioconductor.org/packages/escape/
WGCNA v1.72-1	Langfelder et al.^108^	https://cran.r-project.org/package=WGCNA
hdWGCNA v0.4.03	Morabito et al.^109^	https://smorabit.github.io/hdWGCNA/
ImageJ/Fiji	NIH; Schindelin et al.^110^	https://imagej.net/software/fiji/
Plotly	Plotly	https://plotly.com/python/
ggplot2	Valero-Mora^111^	https://github.com/tidyverse/ggplot2

**Other**		

gentleMACS Dissociator	Miltenyi Biotec	130-093-235
Leica TCS SP8 confocal microscope	Leica Microsystems	N/A

### Experimental model and study participant details

#### Animals

*CX3CR1*^*GFP*^ 3–6-month-old knock-in mice of mixed sex were purchased from Jackson Laboratory (Bar Harbor, ME). The animals were bred and maintained as *CX3CR1*^*GFP*^ heterozygotes on C57BL6 genetic background at the Schepens Eye Research Institute (SERI). The animals were group-housed in a temperature-controlled environment with a standard 12-h dark-light cycle with food and water provided *ad libitum*. All experimental procedures conformed to the National Research Council’s Guide for the Care and Use of Laboratory Animals, the Association for Research in Vision and Ophthalmology (ARVO) guidelines and were approved by the Institutional Animal Care and Use Committee (IACUC) of the SERI.

Here, in the published atlases, and across the manuscript, we focus on preserving metadata and reproducibility. Thus, we use the following terms for highlighting animal sex: Male (PATO:0000384), Female (PATO:0000383), Unknown (unknown), and Mixed (PATO:0002122).

All animal procedures were approved by the Institutional Animal Care and Use Committee (IACUC) of the Schepens Eye Research Institute under protocol #S2680619 and conformed to the National Research Council’s Guide for the Care and Use of Laboratory Animals and ARVO guidelines.

#### Human hESC reporter line

H9-BRN3B:tdTomatoThy1.2 human embryonic stem cells (hESCs) were used to generate donor retinal ganglion cells (RGCs). hESCs were maintained as colonies on six-well plates pre-coated with 2% Matrigel in mTeSR1 medium under low oxygen conditions (3.5% O2, 5% CO2, 95% humidity, 37°C). Medium was changed daily. At approximately 70% confluency, hESCs were passaged with accutase for 8–12 min at room temperature and either split 1:10 to 1:20 or used for differentiation.

### Method details

#### Human stem cell-derived RGC differentiation

H9-BRN3B:tdTomatoThy1.2-human embryonic stem cells (hESCs) were maintained as colonies on six-well plates pre-coated with 2% Matrigel and grown in mTeSR1 medium under low oxygen conditions (3.5% O2, 5% CO2, 95% humidity, 37°C). Medium was changed daily and upon reaching approximately 70% confluency, hESCs were passaged using accutase for 8 to 12 min at room temperature and either split in the ratio of 1:10 to 1:20 or used for differentiation.

Human ESCs were differentiated into 3D retinal organoids as previously described.^6^ Briefly, colonies of hESCs were enzymatically dissociated for 12 min at room temperature into single cells using accutase. To initiate the formation of embryoid bodies (EBs), 7,500 hESCs were seeded per well of an ultra-low attachment U-bottom 96-well plate and cultured under low oxygen condition in mTeSR1 medium supplemented with 20 μM Y-27632. From day 1, differentiation medium was added daily and gradually transitioned from a 1:1 mix of mTeSR1 and BE6.2-NIM [B27 + E6 at 2× concentration – neural induction medium: 1× L-glutamine, 1× sodium pyruvate, 1× antibiotic-antimycotic, 1× B27 without Vitamin A, 0.88 mg/mL NaCl, 38.8 mg/L insulin, 128 mg/L L-ascorbic acid, 28 μg/L selenium, 21.4 mg/L transferrin, and 38.8 mg/L NaHCO3 in DMEM/F12 (3500 mg/L dextrose)] to primarily BE6.2-NIM by day 8. The differentiation medium was supplemented with 9 μM IWR-1-endo and 1% Matrigel from day 1 to day 4 and with 55 ng/mL BMP4 on day 6, to promote neural development early retinal differentiation. On day 8, organoids were transferred in a standard Petri dish and randomly chopped to dissect the optic vesicles and to allow better propagation and greater expansion. To support attachment of organoids and growth of retinal epithelium, the chopped retinal aggregates were transferred in a tissue culture treated T75 flask in Long-Term Retinal Differentiation Medium (LT-RDM) [1× L-glutamine, 1× sodium pyruvate, 1× antibiotic-antimycotic, 1× B27, and 1× non-essential amino acids, 50:50 1× DMEM/F12 (3500 mg/L Dextrose): DMEM (4500 mg/mL dextrose)] supplemented with 10% FBS and 300 nM smoothened agonist (SAG), and cultured under normoxic conditions (20% O2, 5% CO2, 95% humidity, 37°C). LT-RDM supplemented with 300 nM SAG was replaced every other day with a gradually decreasing from 10% FBS to 0% FBS by day 15. On day 15, the clusters of retinal tissue were moved to an ultra-low attachment T75 flask for suspension culture in RDM supplemented with 300 nM SAG. The next day, 2/3 of medium was replaced with LT-RDM supplemented with 300 nM SAG and 2.5% FBS. On day 18, 2/3 of medium was replaced with LT-RDM supplemented with 300 nM SAG and 5% FBS. The medium was replaced every other day with LT-RDM supplemented with 7.5% FBS on day 20 and 10% FBS on day 22 to day 50. LT-RDM was supplemented with 1 mM taurine from day 24–50, with 125 nM all-trans-retinoic acid (ATRA) on day 26, and with 250 nM ATRA from day 28 to day 50. Staring on day 34, LT-RDM was supplemented with 10 μM (2S)-N-[(3,5-Difluorophenyl)acetyl]-L-alanyl-2-phenyl]glycine 1,1-dimethylethyl ester (DAPT). The LT-RDM was replaced every other day until the retinal organoids were dissociated using the Miltenyi Biotec embryoid body dissociation kit according to the manufacturer protocol with the gentleMACS Dissociator protocol (Brain_01 and Brain_02).

Human RGC were sorted by magnetic microbead using the CD90.2 Dynabeads (Miltenyi Biotec, CD90.2 MicroBeads, mouse) according to the manufacturer protocol.

#### *In vivo* transplantation of human ES-derived Retinal ganglion cells

Mice were anesthetized intraperitoneally with 100 mg/kg ketamine hydrochloride and 20 mg/kg xylazine hydrochloride solution diluted with saline and allowed to be fully sedated. Pupils were dilated with 1% tropicamide ophthalmic solution (Baush & Lomb Americas Inc., Bridgewater, NJ). One drop of 0.5% proparacaine hydrochloride ophthalmic solution (Bausch & Lomb Americas Inc., Bridgewater, NJ) was applied onto the surface of the cornea for 2 min. RGCs were prepared in N2B27 medium [50:50 1× DMEM/F12:Neurobasal medium, 1% N2 supplement, 2% B27 supplement, 1× GlutaMAX, and 1× antibiotic antimycotic] with BDNF- and GDNF-loaded PODS (Polyhedrin Delivery System). Using a glass pipette, 1 μL solution containing 15,000 viable tdTomato positive human ES-derived RGCs was injected into the eye subretinally, one eye per mouse. One drop of Neomycin and Polymyxin B Sulfate and Bacitracin Zinc Ophthalmic ointment (Bausch & Lomb Americas Inc., Bridgewater, NJ) was applied to the surface of the injected eye following the procedure.

#### Mouse retina isolation, dissociation, and single-cell suspension preparation

Three days after RGC transplantation, mice were euthanized, injected eyes enucleated and retinal tissue isolated and immediately transferred into a tube containing ice-cold phosphate-buffered saline (PBS) with 0.04% bovine serum albumin (BSA). Eight mouse retinas were pooled to make a sample. The tissue was initially dissociated mechanically, followed by enzymatic method using the Gentle MACS protocol (Miltenyi Biotech, Adult Brain Dissociation Kit, mouse, and rat). Briefly, retinas were allowed to settle to the bottom of the tube and the excess PBS carefully removed with the help of a pipette. The tissues were then rinsed with Hanks’ balanced salt solution (HBSS) and transferred to a MACS M-tube and again left let to settle to the bottom of the tube. Excess HBSS was removed from the MACS tube with the aid of a pipette. The tissues were then resuspended in 2 mL of dissociation solution containing DNAse 1 (1 mg/mL). The tissue-enzyme mixture was incubated at 37°C for 5 min in a heating bath. The M-tube was then rapidly inverted ensuring that all dissociated tissues remained in solution. The tube was then placed into the MACS tissue dissociator and the brain 1 program initiated. At the end of the brain 1 program, the M-tube was removed from the MACS dissociator and incubated at 37°C for 5 min in a heating bath. The tube was then rapidly inverted and placed back into the MACS dissociator and program brain 2 initiated. At the end of the run, 10 mL of isolation buffer was added to the dissociate and the mixture triturated 10 times using a 1000 μL pipette. Then 12 mL of the homogenate was transferred into a 15 mL tube and cells pelleted at 200g for 5 min at 4°C. The resultant cell pellet was resuspended in 500 μL of isolation buffer and filtered through a 70u cell strainer into a FACS collection tube in preparation for cell for sorting.

#### Fluorescent activated cell sorting (FACS)

All tubes were initially pre-blocked with 2% BSA in HBSS. Single stain sample for the labeled cell class, i.e., GFP+, was used to set the gates. Cells were sorted GFP positive only, and collected into collection tubes which contained 200 μL of PBS with 0.04% BSA. The sorted single-cell suspension was immediately used for library preparation for single-cell RNA sequencing.

#### Single-cell RNA library preparation and sequencing

Up to 10,000 viable cells per sample were used for library preparation. Libraries were prepared following the 10× Genomics Chromium Next GEM Single Cell 3ʹ Reagent Kits v3.1 protocol (Chromium Single Cell V(D) J Reagent Kits with Feature Barcoding technology for Cell Surface Protein*,* Document Number CG000186 Rev A, 10× Genomics, (2019, July 25). Briefly, the droplet-based encapsulation method was used to encapsulate the single cells with gel beads, partitioning oil, and a reverse transcription master mix generating gene expression emulsions (GEMs). The generated GEMs were cleaned, expression libraries constructed, barcoded and sequenced at a sequencing depth of 20,000 read pairs per cell, with paired-end dual indexing (See manufactures instructions for details).

#### Quality control and data processing

Raw data was processed using Cell ranger v. 7.0.1. A mouse reference genome was used for sequence alignment (2024-A Mouse GRCm39). Cell ranger output that included matrix, features, and barcodes, was processed using Seurat v. 4.3.0.1.^97^ Quality control metrics included filtering by nCount_RNA, nFeature_RNA, percent.mt, percent.rb. Doublets were removed using the DoubletFinder algorithm.^98^

#### Trajectory reconstruction with RNA velocity

The output of the Cell ranger pipeline including possorted_genome_bam.bam files were used to generate.loom files using velocyto.^99^ Upon the concatenation of the.h5ad FA2-embedded dataset to the.loom splicing matrix, RNA Velocity analysis was performed using scVelo.^101^ The dynamical mode was applied to generate the velocity directions, then upon Leiden clustering the data was subdivided into a ‘homeostatic’ and ‘activated’ cell states based on the directionality of their velocity vectors. Downstream analysis to gain insight into regulatory mechanisms was performed using the two defined population states, i.e., homeostatic (Tmem119^+^) and activated (Apoe^+^) subsets.

#### RNA velocity-based commitment score estimation

To assess commitment of cells within the two identified cell states i.e., homeostatic and activated, and homeostatic populations toward the homeostatic and activated states, cell-level RNA velocity vectors were computed, with data obtained from an integrated single-cell RNA sequencing dataset. Generally, a cell’s velocity vector consists of two components x and y, representing direction and magnitude respectively in a 2D reduced-dimensional ForceAtlas2 embedding.

For each velocity vector *i*, the magnitude is computed as the Euclidean norm:

(Equation 1) $${magnitude}_{i}=\sqrt{v_{x,\mathrm{i}}^{2}+v_{y,\mathrm{i}}^{2}}$$

The direction of each vector *i* is described by an angle computed using the atan2 function:

(Equation 2) $$\theta_{i}=\arctan\;2\left(v_{y,i},v_{x,i}\right)$$

The angle is then converted from radians to degrees and standardized to the range [0, 360):

(Equation 3) $$\theta_{\deg,i}=\left(\frac{\theta_{i}\cdot 180}{\pi }\right)\mathrm{mod}\;360$$

To assess directional bias, the range of angles (0–360°) is discretized into bins. 36 bins of 10° each were used. Once binned, the magnitudes of all velocity vectors falling into the same angular bin are summed. This yields a “cumulative magnitude” per direction bin.

(Equation 4) $$bin\_edges=\left\{0{}^{\circ},\frac{360{}^{\circ}}{N},2\frac{360{}^{\circ}}{N},\ldots ,360{}^{\circ}\right\}$$

(Equation 5) $$b=\mathrm{i}ndex\;such\;that\;\theta_{\deg,i}\in \left[b\mathrm{i}{n\_edges}_{b},bi{n\_edges}_{b+1}\right]$$

(Equation 6) $$M_{bin}\left(b\right)=\sum_{i:b_{i}=b}{magnitude}_{i}$$

Two datasets (mg_tr1 for activated and mg_tr2 for homeostatic subsets) are processed similarly. Their directional cumulative magnitudes were computed. Ratios of cumulative magnitudes were calculated to quantify directional biases or differences in overall velocity distribution between conditions for the angular regions A and B based on the vector directionality. The resultant ratio represents unnormalized commitment score (unCS).

(Equation 7) $$sumA=\sum_{b\in A}M_{bin}\left(b\right);sumB=\sum_{b\in B}M_{bin}\left(b\right)$$

(Equation 8) $$unCS=\frac{sumA}{sumB}\text{,}$$

where unCS >1 shows commitment of mg_tr1 toward homeostatic state.

Since cell commitment depends on cumulative magnitude, which is highly dependent on the number of cells, further normalization is required. Cell numbers can be influenced by either the experimental condition or batch effect between samples or both. Given this, the real commitment score lays between the unnormalized and normalized commitment scores. Normalized commitment score (nCS) is quantified with the number of cells adjustment for populations 1 and 2 as:

(Equation 9) $$nCS=\frac{sumA}{sumB}\cdot \frac{n_{cells}B}{n_{cells}A}$$

#### Assembly of selected publicly available single-cell RNA sequencing datasets for the generation of a mouse reference atlas

To generate a representative mouse reference atlas for efficient mapping of myeloid cell populations from a range of different experimental conditions, we selected publicly available mouse datasets representing health and disease conditions of mice. Each batch in the analysis represents an individual SRR. For the health conditions, we focused on datasets generated from ‘developing mouse retina’, and ‘healthy adult mouse retina’. For the disease conditions, we focused on datasets generated from two mouse models of disease: ‘optic nerve crush model (ONC)’, and the ‘mouse microbead model of glaucoma’. A complete list of the selected datasets is summarized in Table S1. Quality control and data processing was performed independently for each dataset, followed by data integration either by: (1) default Seurat integration, (2) Seurat RPCA-based integration (developing retina), (3) Harmony^102^ (healthy adult retina) or scVI^103^ (optic nerve crush retina). The method of choice for data integration was determined by the number of batches, batch variation, and by the total number of cells. To allow further analysis of myeloid between the conditions, we subset the healthy wild type myeloid population from every condition, as well as from the host upon transplantation myeloid dataset. Each was rescaled, and the subsequent integration was performed using default Seurat integration algorithm. To allow cell-cell interactions analysis and reference mapping of the host upon transplantation myeloid based on the mouse healthy adult retina atlas, we identified the cell classes present in the atlas using the differentially expressed genes and commonly known cell class markers.

#### Healthy adult myeloid trajectory reconstruction

We sampled the myeloid population from the mouse healthy adult retina atlas. Given the number of batches involved, and variation in the number of cells per batch, we applied scVI to generate the mouse healthy adult myeloid subset embedding using 2 hidden layers and 30 latent spaces. Using the scVI-generated embedding, we performed cell fate trajectory reconstruction using scFates^112^ approach. With the reconstructed trajectory, we identified three states of the myeloid cell population. These were: 1) microglia/macrophage, 2) T cells, and 3) dendritic cells. This approach allowed for a more precise reference mapping of our experimental host upon transplantation myeloid population dataset, on to the defined microglia/macrophage myeloid population only. Annotation labels for myeloid cell population upon trajectory reconstruction were added to the healthy adult retina atlas, and the atlas was then used as a reference tool in Azimuth-based^113^ annotation of our host upon transplantation myeloid cell population dataset.

#### *In silico* myeloid replacement and cell-cell interactions analysis

To perform *in silico* myeloid replacement experiments, we used the mouse healthy adult retina to mimic the ‘recipient/host’. To perform the experiment, the integrated dataset was split into three developmental timepoints i.e., young adult, mature adult, and aged, based on the number of weeks postnatal. The software CellChat v. 2.1.0^105^^,^^106^ was used to analyze cell-cell communication, between the cells. The pipeline includes a few non-default settings: trim = 0.1, raw.use = F, population. Size = F, type = ‘truncatedMean” in computeCommunProb(), smoothData using PPI.mouse. Timepoints with low myeloid abundance were skipped to avoid false positives. The myeloid population of the mouse healthy adult retina was replaced in silico with either 1) host myeloid population upon transplantation, or 2) healthy developing retina myeloid cell population. This has been achieved by explicitly subsetting the population of interest from one condition, and merging the remaining part of the dataset with the subsetted microglia/macrophage from the other condition. In both cases, CellChat was used to analyze cell-cell interaction between cells. The resultant three sets of CellChat objects were then explored in a timepoint dependent manner. Using the 20 weeks old time point as proxy for a (6-month-old mouse, one into which RGC would be transplanted *in vivo*) we compared incoming and outgoing signals to the myeloid cells between the adult mouse retina, replacing the myeloid population of host upon transplantation.

The differentially enriched genes were then applied onto the three sets of CellChat objects. For example, a quantitative analysis of Apoe across the three groups (young adult, mature adult, aged) of healthy adult retina where 1) the ‘host’ myeloid is preserved, i.e., native healthy adult, 2) myeloid population is replaced with the host upon transplantation myeloid cell population, 3) myeloid population is replaced with the developing retina myeloid population.

#### ssGSEA pathway analysis of integrated myeloid population

The integrated myeloid population dataset from the 5 conditions (development, adult, glaucoma model, optic nerve crush model, host upon transplantation) were used to perform ssGSEA pathway enrichment analysis using escape v. 1.10.0^107^ We focused our analysis on inflammation-oriented pathways from the Molecular Signature Database. Normalized enrichment score (NES) was averaged per group and pathway, and then normalized from 0 to 1 in a pathway-independent manner. The resulting table of normalized averaged NES in a pathway versus condition was visualized as polygon chart.

#### WGCNA analysis of integrated myeloid population

Furthermore, the integrated myeloid dataset from the 5 conditions described above were used to perform WGCNA analysis using WGCNA v. 1.72–1 and hdWGCNA v. 0.4.03 packages. We identified modules specific to the “host upon transplantation” condition and used the top three with the highest score to build the hub genes network plot. The resulting object were saved in.rds file and these are available upon request.

#### Validation of selected single-cell RNA sequencing targets by immunohistochemistry in retinal whole mounts

To validate the expression of selected differentially expressed genes identified from the analysis, immunohistochemistry analysis was performed on retinal wholemounts after RGC transplantation. Briefly, another batch of mice of the same background, age and sex were transplanted with donor RGC, and on the third day, mice were sacrificed. After enucleation, the globe was punctured with #11 scalpel at the limbus and fixed for 15 min in 4% paraformaldehyde at room temperature. Tissue was washed briefly with PBS to remove excess fixative, followed by removal of anterior segment and lens. Relieving cuts were made to the retina to enable flat-mounting, after which retina was extracted and washed with PBS (pH 7.4; 3 × 5 min) and transferred to blocking solution (PBS with 10% donkey serum, 0.5% Triton-X, 1% BSA). Blocking was performed for 4 h at room temperature, after which retinas were incubated with primary antibodies in blocking solution at 4°C for 3 days. After primary incubation, retinas were washed with PBS (3 × 15 min), then incubated with secondary antibodies in blocking solution for 2 days at 4°C. After secondary incubation, a final series of PBS washes (3 × 15 min) was performed, at which point retinas were flat mounted with Prolong Diamond Antifade Mountant (ThermoFisher Scientific) and cover slipped. Primary antibodies used were: Tmem119 (1:500, Synaptic Systems #400 004), Spp1/OPN (1:500, R&D Systems #AF808), and Apoe (1:500, Synaptic Systems #465 004). Secondary antibodies used were donkey anti-guinea pig 405 and 647 (Jackson Immuno Research #706–475-148 and #706–605-148), and donkey anti-goat 647 (Invitrogen #A32849). Slides were imaged on a Leica TCS SP8 confocal microscope, with optimization of brightness and contrast performed in ImageJ.

#### Microglia quantification and image analysis

Analysis of the microglia images obtained from immunohistochemistry analysis was performed using ImageJ (National Institute of Health). Measurement to the length of microglia based on maximum projection of the z stack images (28.02 μm and 21.23 μm). Length and coordinates of the cells were measured using the “straight” tool in ImageJ. A line was drawn to the cell across the center of the cell touching the longest process on both ends and described as maximum cellular extent. Measurements were made with ImageJ showing the length (in um) and the position (XY coordinates) of the measured cell. Tmem119 and Cx3cr1 were used as markers for microglia state profiling. All the measurements were carried out by 2 investigators in a masked fashion. Quantifications were done according to appropriate counterstains of the markers with DAPI.

To build 3D density plots, the quantification data were imported into R, coerced to numeric, filtered to retain finite values, and spatially cropped to the ImageJ region of interest. The cropped tables were then merged into a single point set. Spatial density was estimated on a regular grid (800 by 800) spanning the cropped field using a two-dimensional Gaussian kernel density estimate (kde2d) with an automatically selected bandwidth (or user-supplied bandwidth when specified). To visualize local mean neurite length, points were first binned to the same grid to compute per-bin counts and summed lengths, then both grids were smoothed with a Gaussian kernel matched to the KDE bandwidth; the smoothed mean length was computed as smoothed summed length divided by smoothed counts (with a small epsilon to avoid division by zero). Finally, a 3D surface plot was generated with Plotly, using the KDE-derived grid as surface height (density) and the smoothed mean length as the surface color.

### Quantification and statistical analysis

Quantitative image analysis was performed in ImageJ/Fiji using maximum-intensity projections of confocal z-stacks. Microglial maximum cellular extent was defined as the longest linear distance across the cell, measured through the cell body between the distal ends of opposing processes. Cell identity and activation state were assigned using the indicated marker combinations, and measurements were independently performed by two investigators masked to marker-defined group identity. In Figures 4L and 4M, *n* represents individual measured cells: Tmem119^+^Spp1^−^, *n* = 34; Tmem119^−^Spp1+, *n* = 78; Cx3cr1^+^ApoE^−^, *n* = 159; and Cx3cr1^+^ApoE^+^, *n* = 38. Statistical significance was defined as *p* < 0.05; the comparisons shown in Figures 4L and 4M yielded *p* < 2.2 × 10ˆ−16ˆ. Exact sample sizes and significance values are provided in the corresponding figure legends. Spatial-density analyses were performed in R after retaining finite measurements and restricting observations to the predefined ImageJ region of interest. Two-dimensional Gaussian kernel-density estimation was performed on an 800 × 800 grid, and local mean cellular extent was calculated from smoothed cell-count and summed-length grids.

Single-cell RNA-sequencing data were processed using Cell Ranger v7.0.1 and Seurat v4.3.0.1. Cells were filtered using library-size, detected-feature, mitochondrial-transcript, and ribosomal-transcript quality-control metrics, and predicted doublets were excluded using DoubletFinder. Public datasets were processed independently before integration using Seurat, reciprocal PCA, Harmony, or scVI, as appropriate for dataset size and batch structure. RNA velocity and trajectory analyses were conducted using velocyto, scVelo, and scFates; cell–cell communication was assessed with CellChat v2.1.0; pathway enrichment was performed with escape v1.10.0; and co-expression networks were analyzed using WGCNA v1.72-1 and hdWGCNA v0.4.03. Normalized enrichment scores were averaged by condition and pathway and rescaled from 0 to 1 for visualization. Timepoints with insufficient myeloid representation and datasets lacking required major retinal cell classes were excluded from relevant CellChat comparisons to reduce unstable or misleading estimates. No formal power calculation was used to determine sample size, and no experimental randomization or stratification was applied; however, image measurements were performed masked. Distributional assumptions were not used for the descriptive single-cell analyses, and finite-value filtering was applied before spatial quantification.

### Additional resources

The host upon transplantation single-cell RNA sequencing data generated in this study is deposited and available on GEO under the GSE285564 reference number.

The code generated in this study was deposited on GitHub: https://github.com/mcrewcow/neurons_transplantation_microglia, and the generated mouse healthy adult retina atlas is available on CELLxGENE: https://cellxgene.cziscience.com/collections/7dd7e8a1-f7c2-4213-9f23-dd52afb7ee16. Researchers interested in contributing data for future versions of the reference atlases should contact the corresponding authors directly. We do not claim any authorship over the original datasets provided in Table S1 and labeled as not published.
