# Supplementary material for: Single-cell transcriptomics reveals reversible microglia/macrophage activation after human retinal neuron transplantation into mouse retina
Source: iScience. 2026 Oct 21;29(11):117232. doi: 10.1016/j.isci.2026.117232 (PMC13718789; doi:10.1016/j.isci.2026.117232)
Supplement: Supplementary file 1 [file mmc1.pdf]

iScience, Volume *29*

Supplemental information

Single-cell transcriptomics reveals reversible microglia/macrophage activation after human retinal neuron transplantation into mouse retina

Emil Kriukov, Anthony Mukwaya, Paul Francis Cullen, Hio Tong Kam, George Baldwin, Volha V. Malechka, Nasrin Refaian, Nikita Bagaev, Everett Labrecque, Sthavir Vinjamuri, Dong Feng Chen, Milica A. Margeta, and Petr Baranov

BD FACSDiva 9.0.1

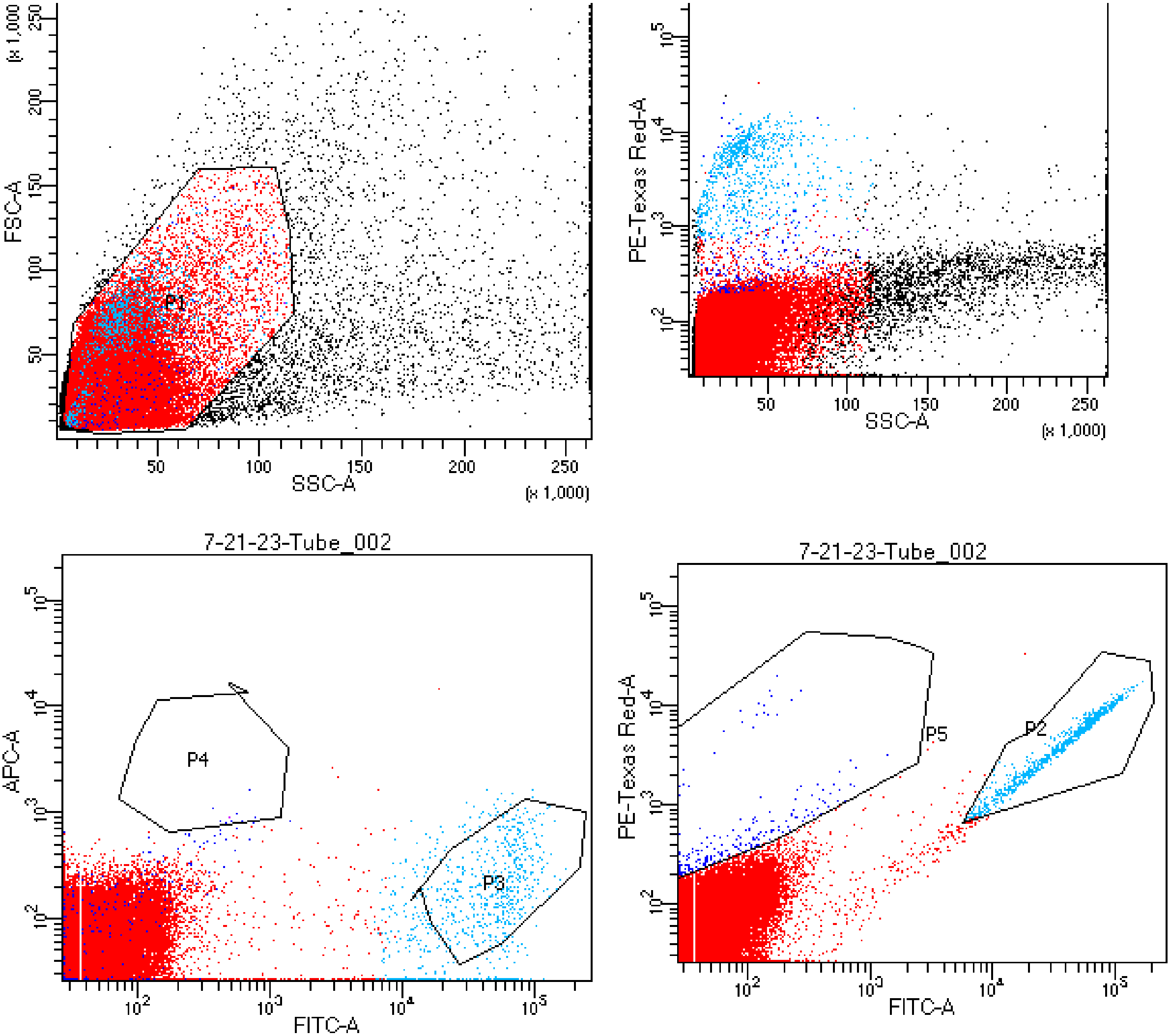

Figure S1. Sorting RGC (TdTomato positive) and Microglia (GFP positive) cells using FACS.

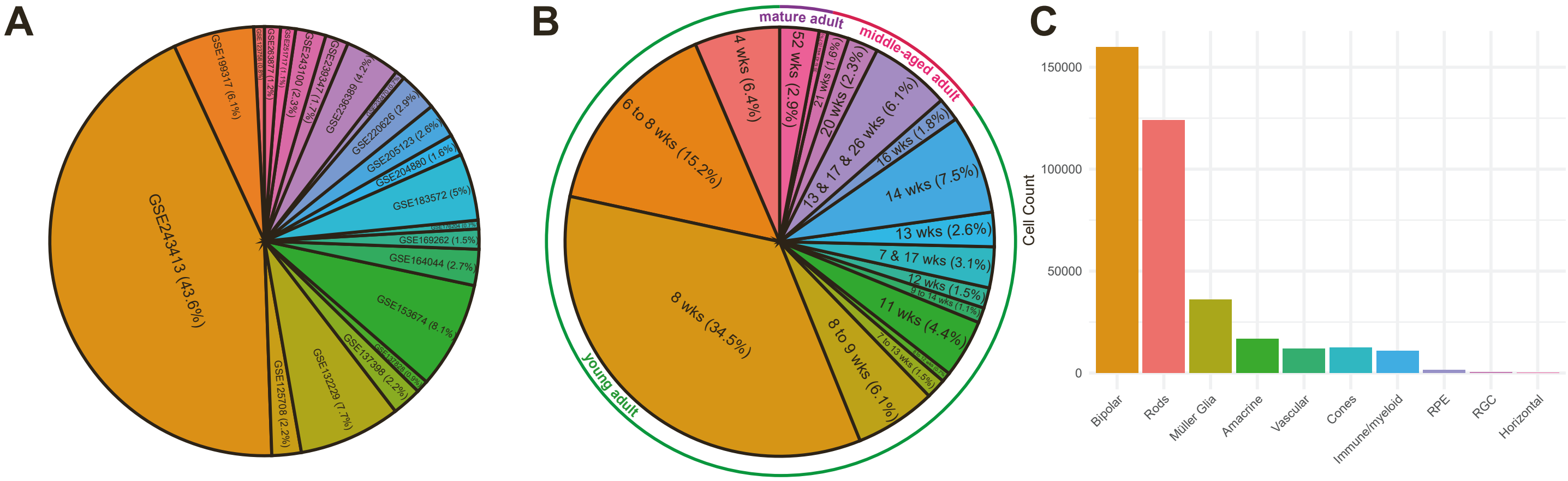
A
GSE243413 (43.6%)
GSE199317 (6.1%)
GSE263877 (1.2%)
GSE257717 (1.1%)
GSE243100 (2.3%)
GSE239347 (1.7%)
GSE236389 (4.2%)
GSE220626 (2.9%)
GSE205123 (2.6%)
GSE204880 (1.6%)
GSE183572 (5%)
GSE169262 (1.5%)
GSE164044 (2.7%)
GSE153674 (8.1%)
GSE137398 (2.2%)
GSE132229 (7.7%)
GSE125708 (2.2%)
B
mature adult
middle-aged adult
young adult
4 wks (6.4%)
52 wks (2.9%)
21 wks (1.6%)
20 wks (2.3%)
13 & 17 & 26 wks (6.1%)
16 wks (1.8%)
14 wks (7.5%)
13 wks (2.6%)
7 & 17 wks (3.1%)
12 wks (1.5%)
9 to 14 wks (1.1%)
11 wks (4.4%)
7 to 13 wks (1.5%)
8 to 9 wks (6.1%)
8 wks (34.5%)
6 to 8 wks (15.2%)
C
Cell Count
150000
100000
50000
0
Bipolar
Rods
Müller Glia
Amacrine
Vascular
Cones
Immune/myeloid
RPE
RGC
Horizontal

Figure S2. Metadata composition of mouse healthy adult retina atlas. (A) Pie chart demonstrating the public datasets contribution into the atlas. (B) Pie chart representing the timeline and timepoints abundance of the atlas. Timepoints are separated into three functional groups: young adult (4 to 14 weeks), mature adult (16 to 21 weeks), aged (rest up to 52 weeks). (C) Barplot highlighting the cell classes abundance in the atlas in the absolute values.

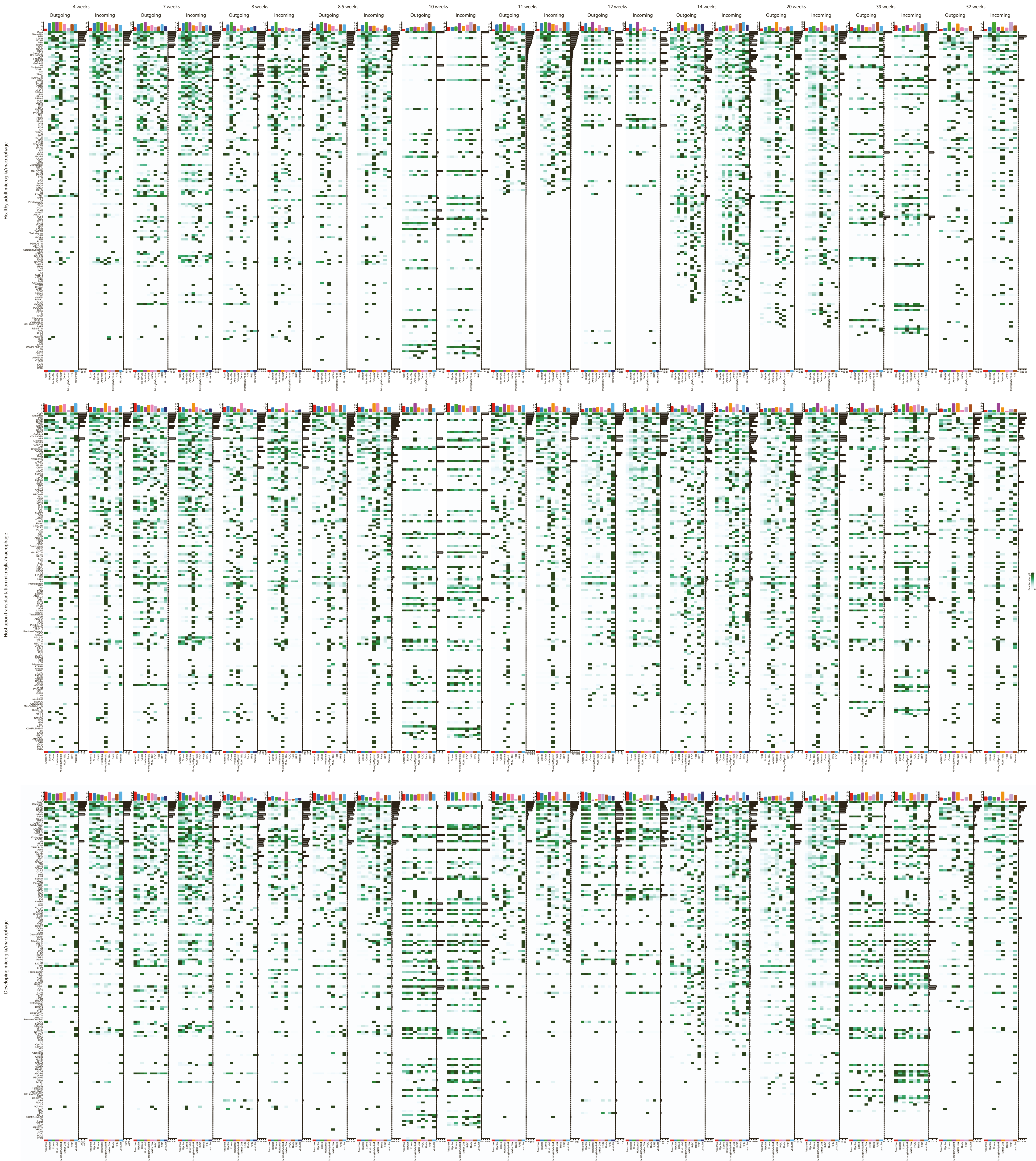
4 weeks
7 weeks
8 weeks
8.5 weeks
10 weeks
11 weeks
12 weeks
14 weeks
20 weeks
39 weeks
52 weeks
Outgoing
Incoming
Healthy adult microglia/macrophage
Host upon transplantation microglia/macrophage
Developing microglia/macrophage

Figure S3. Cell-cell interactions panels for the in silico experiment. Three sets of panels represent three different microglia/macrophage subsets used in the mouse healthy adult retina environment: top – native microglia/macrophage from the healthy adult retina atlas, middle – host upon transplantation microglia/macrophage, bottom – developing microglia/macrophage.

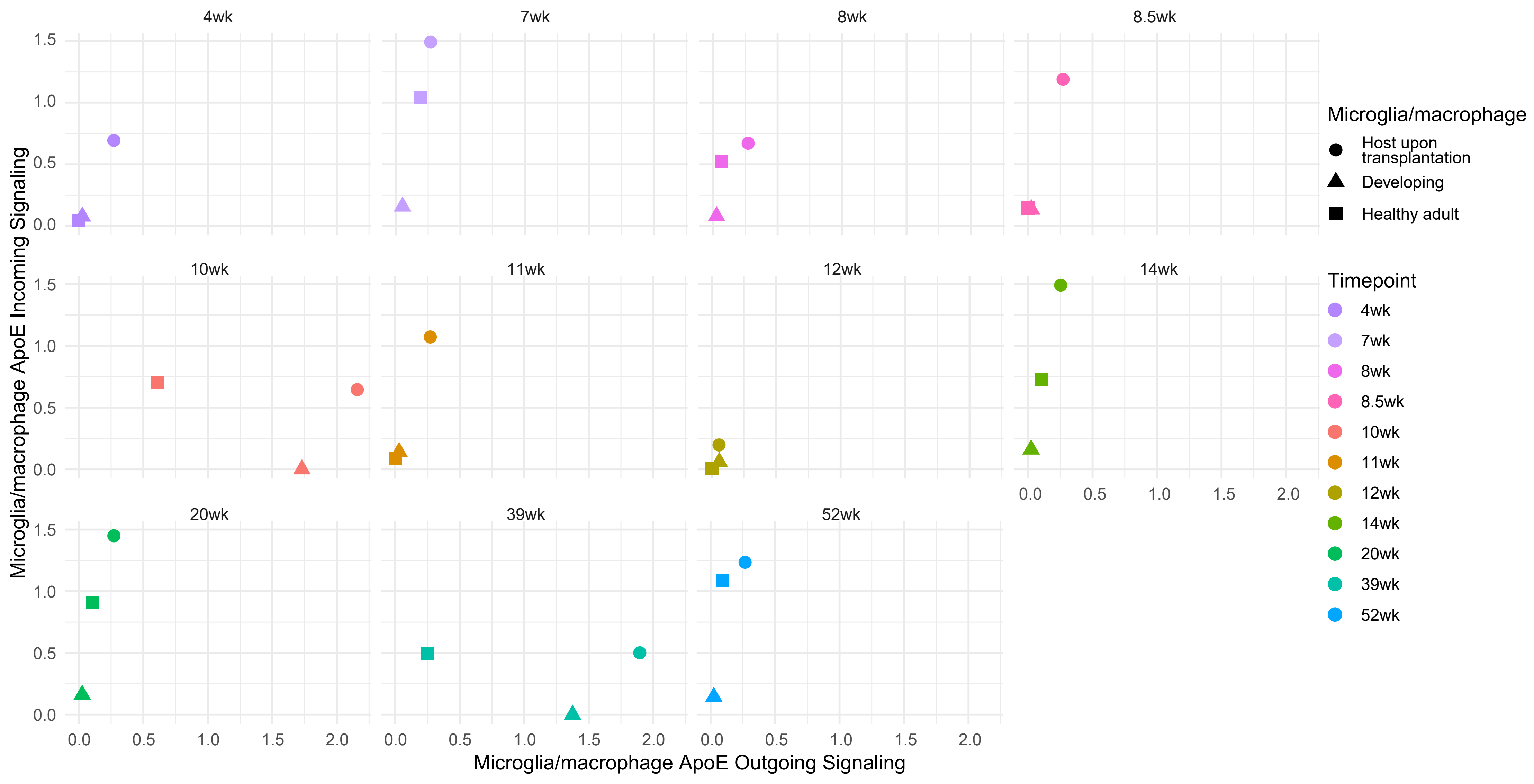
4wk
7wk
8wk
8.5wk
10wk
11wk
12wk
14wk
20wk
39wk
52wk
Microglia/macrophage ApoE Incoming Signaling
Microglia/macrophage ApoE Outgoing Signaling
0.0
0.5
1.0
1.5
2.0
Microglia/macrophage
Host upon transplantation
Developing
Healthy adult
Timepoint
4wk
7wk
8wk
8.5wk
10wk
11wk
12wk
14wk
20wk
39wk
52wk

Figure S4. Apoe signaling strength in cell-cell interactions analysis of three sets of microglia/macrophage.

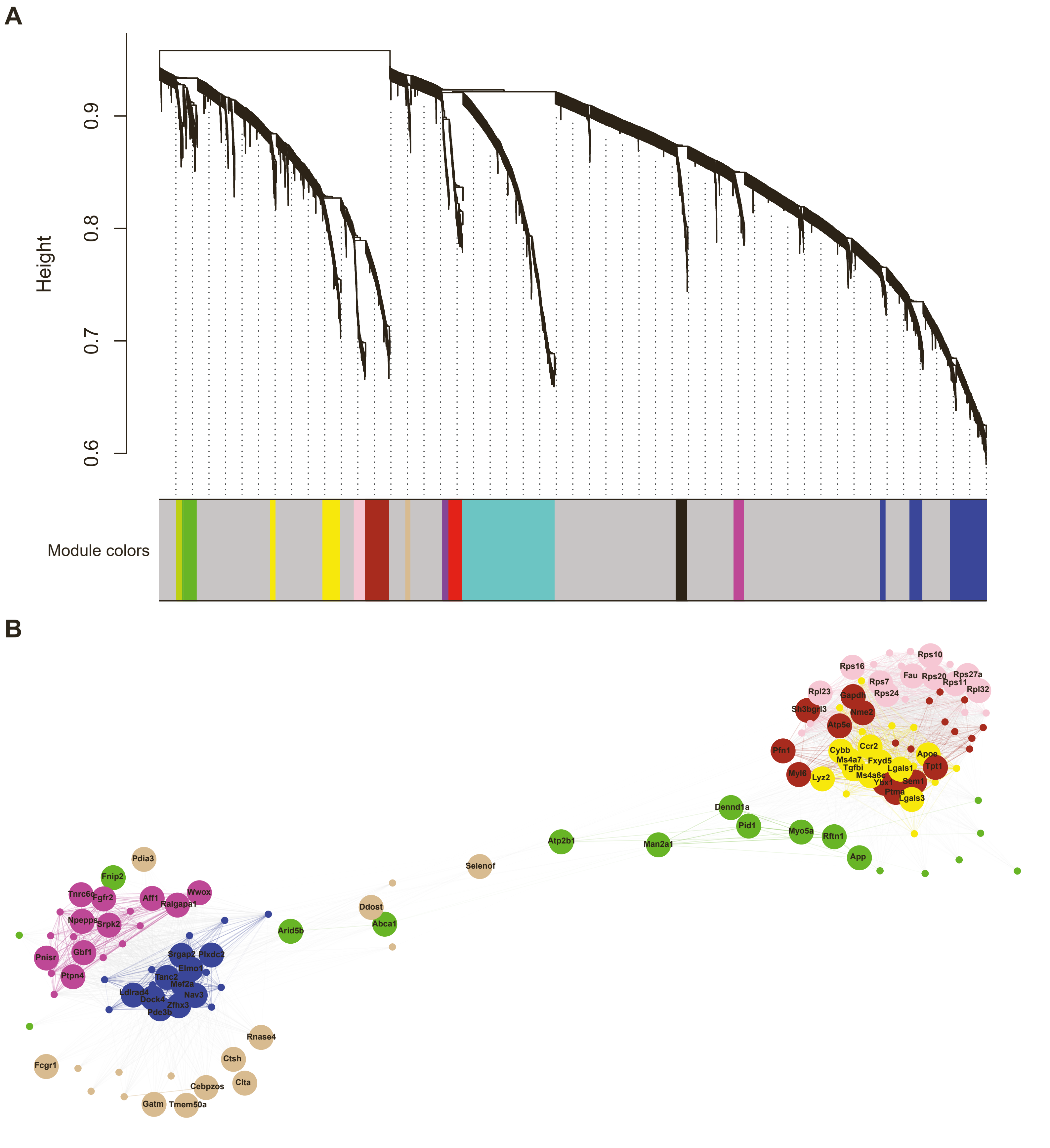
A
Height
0.9
0.8
0.7
0.6
Module colors
B
Rps10
Rps16
Fau
Rps20
Rps27a
Rps7
Rps11
Rpl32
Rpl23
Gapdh
Rps24
Sh3bgrl3
Nme2
Atp5e
Pfn1
Ccr2
Cybb
Apoe
Ms4a7
Fxyd5
Tgfbi
Lgals1
Tpt1
Myl6
Lyz2
Ms4a6c
Ybx1
Sem1
Ptma
Lgals3
Dennd1a
Pid1
Myo5a
Rftn1
Atp2b1
Man2a1
App
Selenof
Pdia3
Fnip2
Wwox
Tnrc6c
Fgfr2
Aff1
Ralgapa1
Npepps
Srpk2
Ddost
Abca1
Arid5b
Gbf1
Srgap2
Plxdc2
Pnisr
Elmo1
Ptpn4
Tanc2
Mef2a
Ldlrad4
Nav3
Dock4
Zfhx3
Pde3b
Rnase4
Ctsh
Fcgr1
Clta
Cebpzos
Gatm
Tmem50a

Figure S5. WGCNA analysis of integrated myeloid dataset. (A) WGCNA dendrogram demonstrating 12 modules discovered for the integrated myeloid dataset. (B) Network analysis of hub genes in the modules with the highest score in host upon transplantation condition demonstrates the modules clustering into two distinct clusters.

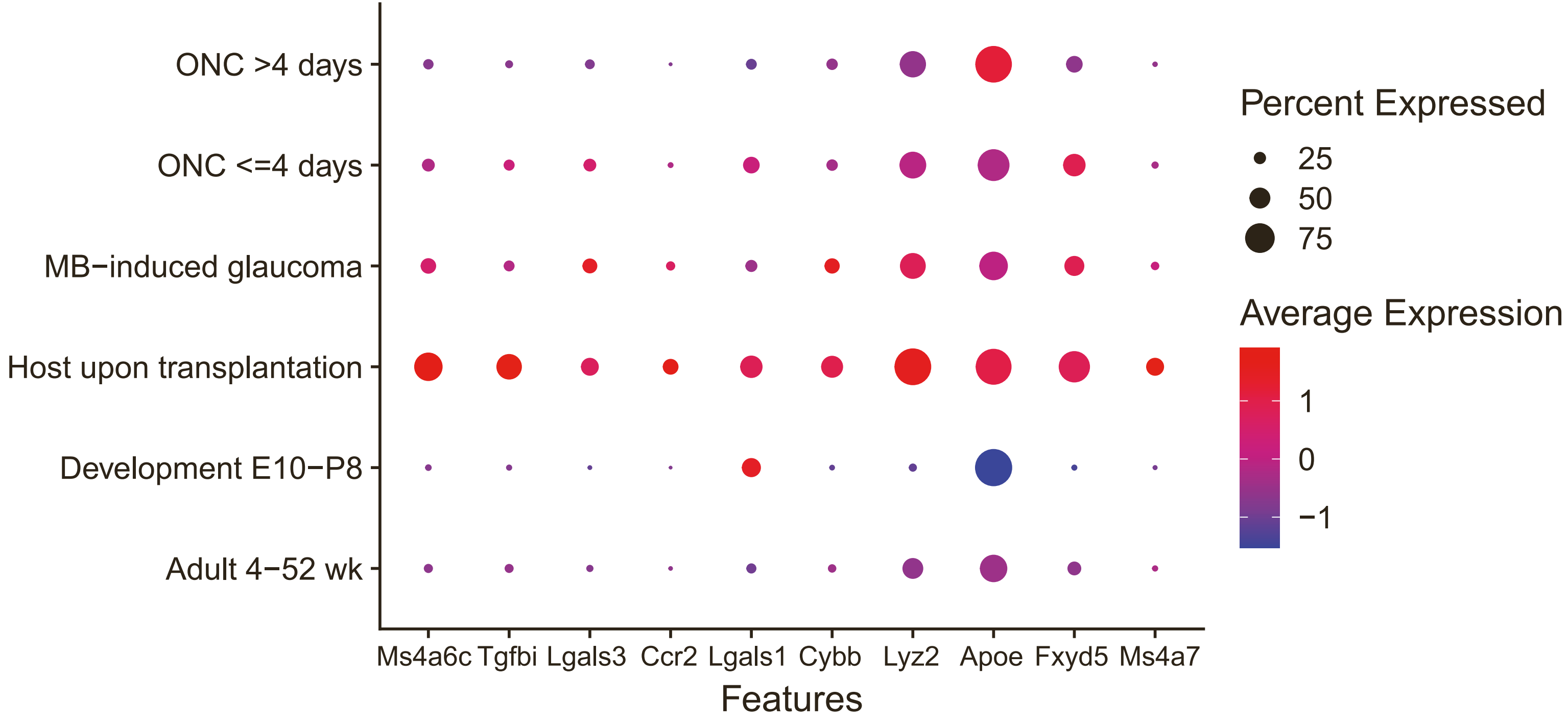
ONC >4 days
ONC <=4 days
MB−induced glaucoma
Host upon transplantation
Development E10−P8
Adult 4−52 wk
Ms4a6c
Tgfbi
Lgals3
Ccr2
Lgals1
Cybb
Lyz2
Apoe
Fxyd5
Ms4a7
Features
Percent Expressed
25
50
75
Average Expression
1
0
−1

Figure S6. Dotplot demonstrating the expression of top hub genes from HUT11 across the conditions in the integrated myeloid dataset.

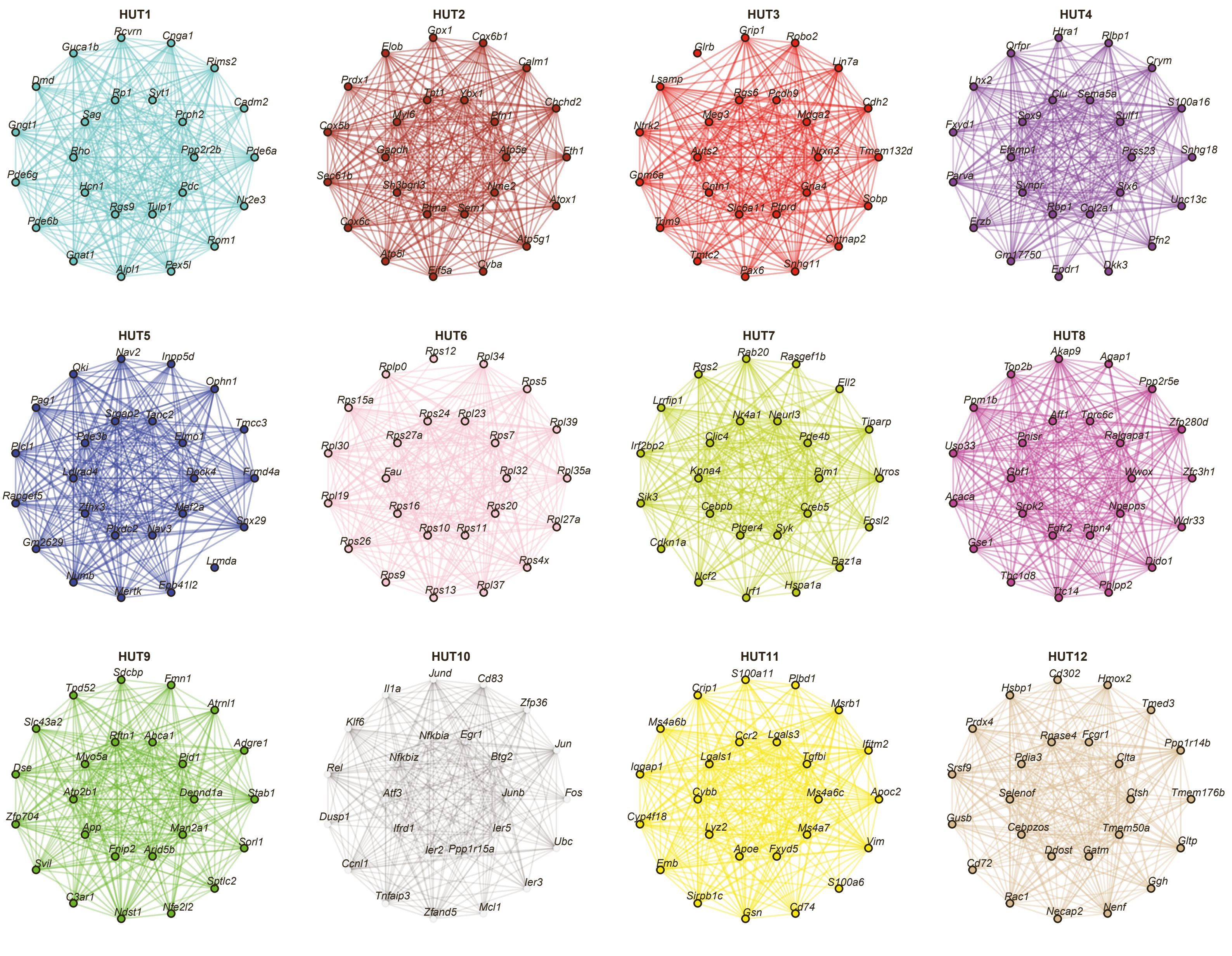
HUT1
Rcvrn
Cnga1
Guca1b
Rims2
Dmd
Rp1
Syt1
Cadm2
Sag
Prph2
Gngt1
Rho
Ppp2r2b
Pde6a
Pde6g
Hcn1
Pdc
Nr2e3
Rgs9
Tulp1
Pde6b
Rom1
Gnat1
Aipl1
Pex5l
HUT2
Gpx1
Cox6b1
Elob
Calm1
Prdx1
Tpt1
Ybx1
Chchd2
Myl6
Pfn1
Cox5b
Gapdh
Atp5e
Fth1
Sec61b
Sh3bgrl3
Nme2
Atox1
Ptma
Sem1
Cox6c
Atp5g1
Atp5l
Eif5a
Cyba
HUT3
Grip1
Robo2
Glrb
Lin7a
Lsamp
Rgs6
Pcdh9
Cdh2
Meg3
Mdga2
Ntrk2
Auts2
Nrxn3
Tmem132d
Gpm6a
Cntn1
Gria4
Sobp
Slc6a11
Ptprd
Trim9
Cntnap2
Tmtc2
Snhg11
Pax6
HUT4
Htra1
Rlbp1
Orfpr
Crym
Lhx2
Clu
Sema5a
S100a16
Fxyd1
Sox9
Sulf1
Efemp1
Prss23
Snhg18
Parva
Synpr
Six6
Unc13c
Rbp1
Col2a1
Frzb
Pfn2
Gm17750
Epdr1
Dkk3
HUT5
Nav2
Inpp5d
Qki
Ophn1
Pag1
Srgap2
Tanc2
Tmcc3
Pde3b
Elmo1
Plcl1
Ldlrad4
Dock4
Frmd4a
Rapgef5
Zfhx3
Mef2a
Plxdc2
Nav3
Snx29
Gm2629
Lrmda
Numb
Mertk
Epb41l2
HUT6
Rps12
Rpl34
Rplp0
Rps5
Rps15a
Rps24
Rpl23
Rpl39
Rps27a
Rps7
Rpl30
Fau
Rpl32
Rpl35a
Rpl19
Rps16
Rps20
Rpl27a
Rps10
Rps11
Rps26
Rps4x
Rps9
Rps13
Rpl37
HUT7
Rab20
Rasgef1b
Rgs2
Ell2
Lrrfip1
Nr4a1
Neurl3
Tiparp
Clic4
Pde4b
Irf2bp2
Kcna4
Pim1
Nrros
Sik3
Cebpb
Creb5
Fosl2
Ptger4
Syk
Cdkn1a
Baz1a
Ncf2
Irf1
Hspa1a
HUT8
Akap9
Top2b
Agap1
Ppp2r5e
Ppm1b
Aff1
Tnrc6c
Zfp280d
Pnisr
Ralgapa1
Usp33
Gbf1
Wwox
Zfc3h1
Acaca
Srpk2
Npepps
Wdr33
Fgfr2
Ptpn4
Gse1
Dido1
Tbc1d8
Ttc14
Phlpp2
HUT9
Sdcbp
Fmn1
Tpd52
Atrnl1
Slc43a2
Rftn1
Abca1
Adgre1
Myo5a
Pid1
Dse
Atp2b1
Dennd1a
Stab1
Zfp704
App
Man2a1
Sorl1
Fnip2
Arid5b
Svil
Sptlc2
C3ar1
Ndst1
Nfe2l2
HUT10
Jund
Il1a
Cd83
Zfp36
Klf6
Nfkbia
Egr1
Jun
Nfkbiz
Btg2
Rel
Atf3
Junb
Fos
Dusp1
Ifrd1
Ier5
Ier2
Ppp1r15a
Ubc
Ccnl1
Ier3
Tnfaip3
Zfand5
Mcl1
HUT11
S100a11
Plbd1
Crip1
Msrb1
Ms4a6b
Ccr2
Lgals3
Ifitm2
Lgals1
Tgfbi
Iqgap1
Cybb
Ms4a6c
Apoc2
Cyp4f18
Lyz2
Ms4a7
Vim
Apoe
Fxyd5
Emb
S100a6
Sirpb1c
Gsn
Cd74
HUT12
Cd302
Hsbp1
Hmox2
Tmed3
Prdx4
Rnase4
Fcgr1
Ppp1r14b
Pdia3
Clta
Srsf9
Selenof
Ctsh
Tmem176b
Gusb
Cebpzos
Tmem50a
Gltp
Ddost
Gatm
Cd72
Ggh
Rac1
Necap2
Nenf

Figure S7. Top 25 genes in each module generated through WGCNA analysis of integrated myeloid dataset.

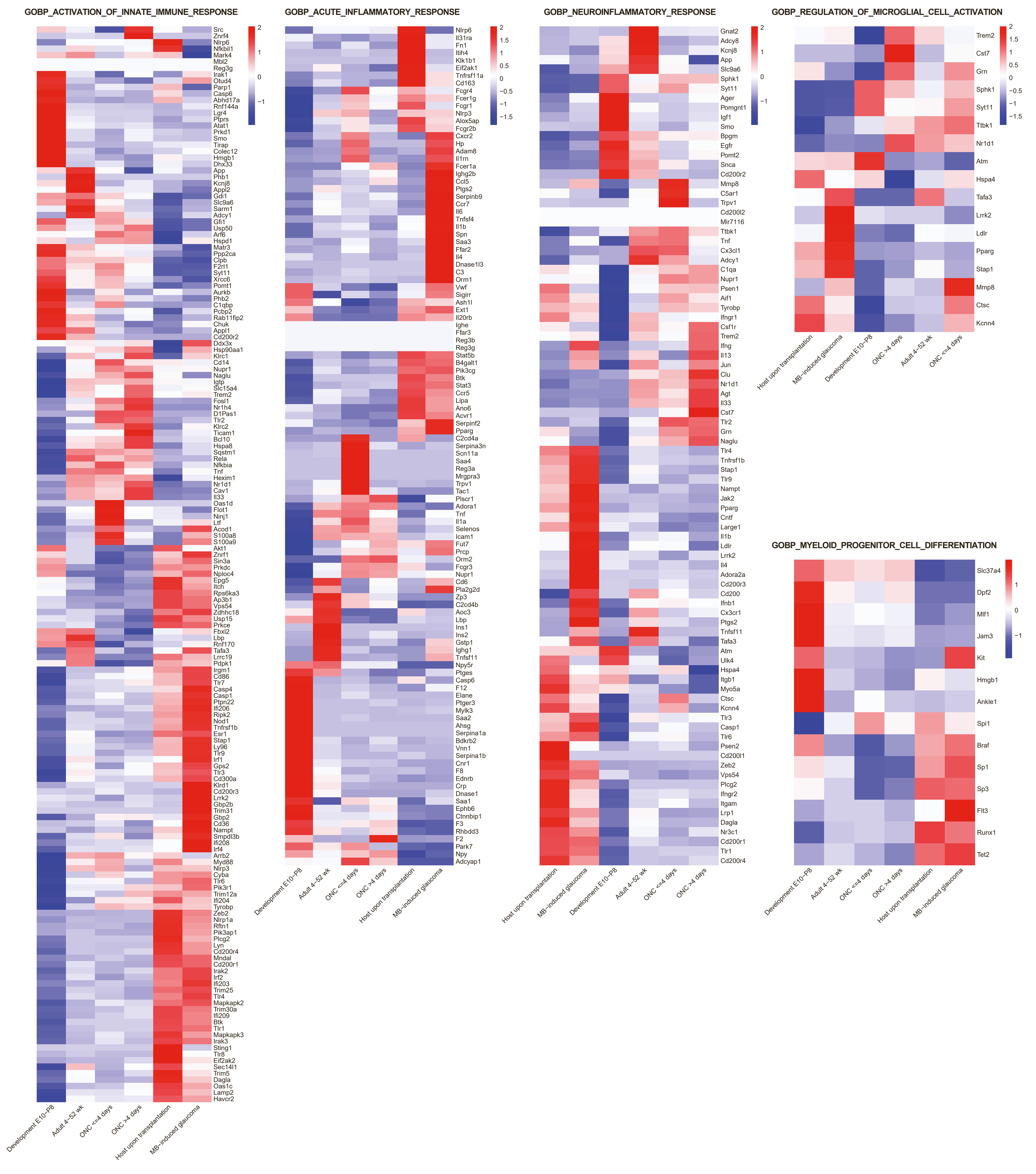
GOBP_ACTIVATION_OF_INNATE_IMMUNE_RESPONSE
2
1
0
−1
Src
Znrf4
Nlrp6
Nfkbil1
Mark4
Mbl2
Reg3g
Irak1
Otud4
Parp1
Casp6
Abhd17a
Rnf144a
Lgr4
Ptprs
Atat1
Prkd1
Smo
Tirap
Colec12
Hmgb1
Dhx33
App
Phb1
Kcnj8
Appl2
Gdi1
Slc9a6
Sarm1
Adcy1
Gfi1
Usp50
Arf6
Hspd1
Matr3
Ppp2ca
Clpb
F2rl1
Syt11
Xrcc6
Pomt1
Aurkb
Phb2
C1qbp
Pcbp2
Rab11fip2
Chuk
Appl1
Cd200r2
Ddx3x
Hsp90aa1
Klrc1
Cd14
Nupr1
Naglu
Igtp
Slc15a4
Trem2
Fosl1
Nr1h4
D1Pas1
Tlr2
Klrc2
Ticam1
Bcl10
Hspa8
Sqstm1
Rela
Nfkbia
Tnf
Hexim1
Nr1d1
Cav1
Il33
Oas1d
Flot1
Ninj1
Ltf
Acod1
S100a8
S100a9
Akt1
Znrf1
Sin3a
Prkdc
Nploc4
Epg5
Itch
Rps6ka3
Ap3b1
Vps54
Zdhhc18
Usp15
Prkce
Fbxl2
Lbp
Rnf170
Tafa3
Lrrc19
Pdpk1
Irgm1
Cd86
Tlr7
Casp4
Casp1
Ptpn22
Ifi206
Ripk2
Nod1
Tnfrsf1b
Esr1
Stap1
Ly96
Tlr9
Irf1
Gps2
Tlr3
Cd300a
Klrd1
Cd200r3
Lrrk2
Gbp2b
Trim31
Gbp2
Cd36
Nampt
Smpdl3b
Ifi208
Irf4
Arrb2
Myd88
Nlrp3
Cyba
Tlr6
Pik3r1
Trim12a
Ifi204
Tyrobp
Zeb2
Nlrp1a
Rftn1
Pik3ap1
Plcg2
Lyn
Cd200r4
Mndal
Cd200r1
Irak2
Irf2
Ifi203
Trim25
Tlr4
Mapkapk2
Trim30a
Ifi209
Btk
Tlr1
Mapkapk3
Irak3
Sting1
Tlr8
Eif2ak2
Sec14l1
Trim5
Dagla
Oas1c
Lamp2
Havcr2
Development E10–P8
Adult 4–52 wk
ONC <=4 days
ONC >4 days
Host upon transplantation
MB–induced glaucoma
GOBP_ACUTE_INFLAMMATORY_RESPONSE
2
1.5
1
0.5
0
−0.5
−1
−1.5
Nlrp6
Il31ra
Fn1
Itih4
Klk1b1
Eif2ak1
Tnfrsf11a
Cd163
Fcgr4
Fcer1g
Fcgr1
Nlrp3
Alox5ap
Fcgr2b
Cxcr2
Hp
Adam8
Il1rn
Fcer1a
Ighg2b
Ccl5
Ptgs2
Serpinb9
Ccr7
Il6
Tnfsf4
Il1b
Spn
Saa3
Ffar2
Il4
Dnase1l3
C3
Orm1
Vwf
Sigirr
Ash1l
Ext1
Il20rb
Ighe
Ffar3
Reg3b
Reg3g
Stat5b
B4galt1
Pik3cg
Btk
Stat3
Ccr5
Lipa
Ano6
Acvr1
Serpinf2
Pparg
C2cd4a
Serpina3n
Scn11a
Saa4
Reg3a
Mrgpra3
Trpv1
Tac1
Plscr1
Adora1
Tnf
Il1a
Selenos
Icam1
Fut7
Prcp
Orm2
Fcgr3
Nupr1
Cd6
Pla2g2d
Zp3
C2cd4b
Aoc3
Lbp
Ins1
Ins2
Gstp1
Ighg1
Tnfsf11
Npy5r
Ptges
Casp6
F12
Elane
Ptger3
Mylk3
Saa2
Ahsg
Serpina1a
Bdkrb2
Vnn1
Serpina1b
Cnr1
F8
Ednrb
Crp
Dnase1
Saa1
Ephb6
Ctnnbip1
F3
Rhbdd3
F2
Park7
Npy
Adcyap1
Development E10–P8
Adult 4–52 wk
ONC <=4 days
ONC >4 days
Host upon transplantation
MB–induced glaucoma
GOBP_NEUROINFLAMMATORY_RESPONSE
2
1
0
−1
Gnat2
Adcy8
Kcnj8
App
Slc9a6
Sphk1
Syt11
Ager
Pomgnt1
Igf1
Smo
Bpgm
Egfr
Pomt2
Snca
Cd200r2
Mmp8
C5ar1
Trpv1
Cd200l2
Mir7116
Ttbk1
Tnf
Cx3cl1
Adcy1
C1qa
Nupr1
Psen1
Aif1
Tyrobp
Ifngr1
Csf1r
Trem2
Ifng
Il13
Jun
Clu
Nr1d1
Agt
Il33
Cst7
Tlr2
Grn
Naglu
Tlr4
Tnfrsf1b
Stap1
Tlr9
Nampt
Jak2
Pparg
Cntf
Large1
Il1b
Ldlr
Lrrk2
Il4
Adora2a
Cd200r3
Cd200
Ifnb1
Cx3cr1
Ptgs2
Tnfsf11
Tafa3
Atm
Ulk4
Hspa4
Itgb1
Myo5a
Ctsc
Kcnn4
Tlr3
Casp1
Tlr6
Psen2
Cd200l1
Zeb2
Vps54
Plcg2
Ifngr2
Itgam
Lrp1
Dagla
Nr3c1
Cd200r1
Tlr1
Cd200r4
Host upon transplantation
MB–induced glaucoma
Development E10–P8
Adult 4–52 wk
ONC <=4 days
ONC >4 days
GOBP_REGULATION_OF_MICROGLIAL_CELL_ACTIVATION
2
1.5
1
0.5
0
−0.5
−1
−1.5
Trem2
Cst7
Grn
Sphk1
Syt11
Ttbk1
Nr1d1
Atm
Hspa4
Tafa3
Lrrk2
Ldlr
Pparg
Stap1
Mmp8
Ctsc
Kcnn4
Host upon transplantation
MB–induced glaucoma
Development E10–P8
ONC >4 days
Adult 4–52 wk
ONC <=4 days
GOBP_MYELOID_PROGENITOR_CELL_DIFFERENTIATION
1
0
−1
Slc37a4
Dpf2
Mlf1
Jam3
Kit
Hmgb1
Ankle1
Spi1
Braf
Sp1
Sp3
Flt3
Runx1
Tet2
Development E10–P8
Adult 4–52 wk
ONC <=4 days
ONC >4 days
Host upon transplantation
MB–induced glaucoma

Figure S8. Heatmap highlighting the expression patterns of the genes belonging to the pathways studied in the integrated myeloid dataset.

Supplementary Table 1.

GSE	DOI	Condition
GSE123758	Not published_3, as in CELLxGENE	Mouse healthy adult retina atlas
GSE125708[86]	10.1073/pnas.1821122116 (https://pubmed.ncbi.nlm.nih.gov/30988181/)	Mouse healthy adult retina atlas
GSE132229[87]	10.1073/pnas.1915571116 (https://pubmed.ncbi.nlm.nih.gov/31843893/)	Mouse healthy adult retina atlas
GSE137398[26]	10.1016/j.neuron.2019.11.006 (https://pubmed.ncbi.nlm.nih.gov/31784286/)	Mouse healthy adult retina atlas
GSE137828[26]	10.1016/j.neuron.2019.11.006 (https://pubmed.ncbi.nlm.nih.gov/31784286/)	Mouse healthy adult retina atlas
GSE153674[88]	PMID: 33088174 (https://pubmed.ncbi.nlm.nih.gov/33088174/)	Mouse healthy adult retina atlas
GSE164044[89]	10.1016/j.neuron.2019.08.002 (https://pubmed.ncbi.nlm.nih.gov/31493975/)	Mouse healthy adult retina atlas
GSE169262[90]	10.1038/s41467-021-27924-y (https://pubmed.ncbi.nlm.nih.gov/35017532/)	Mouse healthy adult retina atlas
GSE176204[36]	10.1016/j.celrep.2023.112889 (https://pubmed.ncbi.nlm.nih.gov/37527036/)	Mouse healthy adult retina atlas
GSE183572[91]	10.1038/s42003-022-03676-3 (https://www.nature.com/articles/s42003-022-03676-3)	Mouse healthy adult retina atlas
GSE199317[92]	10.1038/s41590-023-01437-w (https://pubmed.ncbi.nlm.nih.gov/36807640/)	Mouse healthy adult retina atlas
GSE204880[93]	10.1038/s42003-023-05300-4 (https://pubmed.ncbi.nlm.nih.gov/37670124/)	Mouse healthy adult retina atlas
GSE205123[94]	10.1016/j.ygeno.2023.110644 (https://pubmed.ncbi.nlm.nih.gov/37279838/)	Mouse healthy adult retina atlas
GSE220626[95]	10.3389/fcell.2023.1252547 (https://pubmed.ncbi.nlm.nih.gov/37691820/)	Mouse healthy adult retina atlas
GSE232470[96]	10.1186/s40478-023-01580-3 (https://pubmed.ncbi.nlm.nih.gov/37226256/)	Mouse healthy adult retina atlas
GSE236389	Not published_1, as in CELLxGENE	Mouse healthy adult retina atlas
GSE239347[97]	10.1073/pnas.2314698120 (https://pubmed.ncbi.nlm.nih.gov/38064509/)	Mouse healthy adult retina atlas
GSE243100[98]	10.2337/db23-0368 (https://pubmed.ncbi.nlm.nih.gov/37976214/)	Mouse healthy adult retina atlas
GSE243413[30]	10.1016/j.isci.2024.109916 (https://pubmed.ncbi.nlm.nih.gov/38812536/)	Mouse healthy adult retina atlas
GSE251717[99]	10.1101/2024.03.18.585452 (https://pubmed.ncbi.nlm.nih.gov/38562864/)	Mouse healthy adult retina atlas
GSE263877[100]	10.1038/s41467-024-50033-5 (https://pubmed.ncbi.nlm.nih.gov/39009597/)	Mouse healthy adult retina atlas
GSE137398[26]	10.1016/j.neuron.2019.11.006 (https://pubmed.ncbi.nlm.nih.gov/31784286/)	Mouse optic nerve crush retina
GSE199317[92]	10.1038/s41590-023-01437-w (https://pubmed.ncbi.nlm.nih.gov/36807640/)	Mouse optic nerve crush retina
GSE201254[101]	10.1016/j.neuron.2022.06.002 (https://pubmed.ncbi.nlm.nih.gov/35767994/)	Mouse optic nerve crush retina
GSE206622[101]	10.1016/j.neuron.2022.06.022 (https://pubmed.ncbi.nlm.nih.gov/35952672/)	Mouse optic nerve crush retina
GSE206623[101]	10.1016/j.neuron.2022.06.022 (https://pubmed.ncbi.nlm.nih.gov/35952672/)	Mouse optic nerve crush retina
GSE206624[101]	10.1016/j.neuron.2022.06.022 (https://pubmed.ncbi.nlm.nih.gov/35952672/)	Mouse optic nerve crush retina
GSE206625[101]	10.1016/j.neuron.2022.06.022 (https://pubmed.ncbi.nlm.nih.gov/35952672/)	Mouse optic nerve crush retina
GSE223530[102]	10.1073/pnas.2305947121 (https://pubmed.ncbi.nlm.nih.gov/38289952/)	Mouse optic nerve crush retina
GSE228627[103]	10.1101/2023.11.17.567614 (https://pubmed.ncbi.nlm.nih.gov/38014168/)	Mouse optic nerve crush retina
GSE232470[96]	10.1186/s40478-023-01580-3 (https://pubmed.ncbi.nlm.nih.gov/37226256/)	Mouse optic nerve crush retina
GSE241268[104]	10.1038/s41467-024-46428-z (https://pubmed.ncbi.nlm.nih.gov/38467611/)	Mouse optic nerve crush retina
GSE248537[104]	10.1038/s41467-024-46428-z (https://pubmed.ncbi.nlm.nih.gov/38467611/)	Mouse optic nerve crush retina

GSE	DOI	Condition
GSE226456[36]	10.1016/j.celrep.2023.112889 (https://pubmed.ncbi.nlm.nih.gov/37527036/)	Mouse MB-induced glaucoma retina
GSE247776[105]	10.1038/s41467-024-50050-4 (https://pubmed.ncbi.nlm.nih.gov/39080269/)	Mouse MB-induced glaucoma retina
not published	not published, as in CELLxGENE (Dong Feng Chen Lab)	Mouse MB-induced glaucoma retina
GSE118614[106]	10.1016/j.neuron.2019.04.010 (https://pubmed.ncbi.nlm.nih.gov/31128945/)	Mouse healthy developing retina
GSE122466[107]	10.1242/dev.178103 (https://pubmed.ncbi.nlm.nih.gov/31399471/)	Mouse healthy developing retina
GSE139904[108]	10.1126/sciadv.abj9846 (https://pubmed.ncbi.nlm.nih.gov/34757798/)	Mouse healthy developing retina
GSE186069[109]	10.1038/s41586-024-07069-w (https://pubmed.ncbi.nlm.nih.gov/38947520/)	Mouse healthy developing retina
GSE172101[110]	10.1242/dev.199399 (https://pubmed.ncbi.nlm.nih.gov/34143204/)	Mouse healthy developing retina
GSE202734[111]	10.1016/j.celrep.2023.112237 (https://pubmed.ncbi.nlm.nih.gov/36924502/)	Mouse healthy developing retina
GSE222956[111]	10.1016/j.celrep.2023.112237 (https://pubmed.ncbi.nlm.nih.gov/36924502/)	Mouse healthy developing retina
GSE228590[109]	10.1038/s41586-024-07069-w (https://pubmed.ncbi.nlm.nih.gov/38947520/)	Mouse healthy developing retina
GSE245542[112]	10.1016/j.celrep.2024.114291 (https://pubmed.ncbi.nlm.nih.gov/38823017/)	Mouse healthy developing retina
GSE252861[113]	10.1016/j.isci.2024.110100 (https://pubmed.ncbi.nlm.nih.gov/38947520/)	Mouse healthy developing retina
